# A software pipeline for medical information extraction with large language models, open source and suitable for oncology

**DOI:** 10.1038/s41698-025-01103-4

**Published:** 2025-09-17

**Authors:** Isabella Catharina Wiest, Fabian Wolf, Marie-Elisabeth Leßmann, Marko van Treeck, Dyke Ferber, Jiefu Zhu, Heiko Boehme, Keno K. Bressem, Hannes Ulrich, Matthias P. Ebert, Jakob Nikolas Kather

**Affiliations:** 1 Department of Medicine II, Medical Faculty Mannheim, Heidelberg University, Mannheim, Germany; 2 Else Kroener Fresenius Center for Digital Health, Faculty of Medicine and University Hospital Carl Gustav Carus, TUD Dresden University of Technology, Dresden, Germany; 3 Department of Medicine I, Faculty of Medicine and University Hospital Carl Gustav Carus, TUD Dresden University of Technology, Dresden, Germany; 4 Medical Oncology, National Center for Tumor Diseases (NCT), University Hospital Heidelberg, Heidelberg, Germany; 5 National Center for Tumor Diseases (NCT), NCT/UCC Dresden, a partnership between DKFZ, Faculty of Medicine and University Hospital Carl Gustav Carus, TUD Dresden University of Technology, and Helmholtz-Zentrum Dresden-Rossendorf (HZDR), Dresden, Germany; 6 Department of Cardiovascular Radiology and Nuclear Medicine, Technical University of Munich, School of Medicine and Health, German Heart Center, TUM University Hospital, Munich, Germany; 7 Institute for Medical Informatics and Statistics, Kiel University and University Hospital Schleswig-Holstein, Campus Kiel, Schleswig-Holstein Germany; 8 DKFZ Hector Cancer Institute at the University Medical Center, Mannheim, Germany

**Keywords:** Cancer, Translational research, Cancer

## Abstract

In medical oncology, text data, such as clinical letters or procedure reports, is stored in an unstructured way, making quantitative analysis difficult. Manual review or structured information retrieval is time-consuming and costly, whereas Large Language Models (LLMs) offer new possibilities in natural language processing for structured Information Extraction (IE) from medical free text. This protocol describes a workflow (LLM-AIx) for extracting predefined clinical entities from unstructured oncology text using privacy-preserving LLMs. It addresses a key barrier in clinical research and care by enabling efficient information extraction to support decision-making and large-scale data analysis. It runs on local hospital infrastructure, eliminating the need to transfer patient data externally. We demonstrate its utility on 100 pathology reports from The Cancer Genome Atlas (TCGA) for TNM stage extraction. LLM-AIx requires no programming skills and offers a user-friendly interface for rapid, structured data extraction from clinical free text.

## Introduction

Medical free text contains essential information, such as details about patient characteristics and therapy course and maps the patient journey substantially better than structured medical information from electronic health records (EHRs) alone^1–3^. This medical free text contains the main reasoning as well as observations from medical staff within a variety of different report types, such as clinical letters as well as documentation of different diagnostic and therapeutic procedures^4^. In its unstructured form, text is not available for quantitative analysis and is therefore not accessible for research, quality analysis or interoperable data exchange^5^. Forcing medical staff into structured documentation, however, is not feasible due to time constraints and shortage of personnel in the healthcare system. This leads to an increasing documentation burden and decreases the time available for actual patient care^6^. Therefore, systematically extracting information from free text is crucial for the medical field: It enables researchers to investigate rare diseases^7^, allows better tracking, overview, and exchange of patient information among different inpatient and outpatient providers via a comprehensive health record, and systematic quality control assessment^8,9^.

Previous methods to mine medical free text fall short because they are either not capable of processing large amounts of text and have limited capabilities to grasp context, or need task-specific fine-tuning^10^, whereas our method solely relies on in-context learning of large language models. In-context-learning enhances LLMs’ performance on new tasks by using examples or step-by-step instructions within the prompt^11,12^. Additionally, narrative medical text comes from various source systems, which complicates a streamlined processing. Some reports may only be accessible in portable document format (PDF) from the clinical information system (CIS), others originate from secondary software in a variety of different formats^13^. Data transformation processes to harmonize all the data formats from their source systems within one central database are not ubiquitously established^14^. We therefore present an open-source, LLM-based pipeline which tackles these challenges in medical information extraction (IE). Additionally, our pipeline extracts structured information elements that can be flexibly defined by the user. This is advantageous compared to traditional IE, where predefined categories and relationships are extracted. Our approach offers a highly flexible process for handling large-scale unstructured data^15^.

Our central contribution is a lightweight yet complete pipeline that integrates local model deployment, data preprocessing, IE, and automated evaluation within an intuitive interface, offering the necessary functionality for efficient clinical IE without unnecessary complexity. It is able to transform various types of unstructured medical text data, such as clinical notes, procedure reports or entire clinical letters, into structured CSV format, suitable for quantitative analysis. This development was motivated by the need for a scalable solution that accommodates the technical expertise and deep medical domain understanding required for effective data utilization in healthcare. The method has been developed, applied and tested for several use cases, namely extracting suicidality of psychiatric admission notes^16^, tested with different LLMs from Meta AI (Llama-2 models). Additionally, we extracted several symptoms and diagnoses for detection of decompensated liver cirrhosis from emergency room (ER) admission notes^17^. Furthermore, we applied the pipeline for extracting adverse events from endoscopy reports of endoscopic mucosal resection and colonoscopies^18^. All of these proof-of-concept studies led to the development of the entire pipeline presented here, which comprise an intuitive graphical user interface (GUI), data preprocessing, LLM-based IE as well as automated evaluation of the process within one pipeline. Previously, we introduced the LLM Anonymizer, which is a special case for IE with the purpose of anonymizing medical reports^19^.

The latest open-source LLMs can easily be implemented within the pipeline, which also facilitates benchmarking of different models in accurately extracting relevant entities and information based on the specific needs of requestors. Currently, all models available in Generative Pre-Trained Transformers (GPT)- Generated Unified Format (GGUF) can be included in the pipeline, such as Llama-2 with 7 billion parameters (7b), Llama-2 70b, Llama-3.1 8b, Llama-3.2 70b, Llama-2 “Sauerkraut” 70b, Phi-3, Mistral 7b and many more. By producing outputs in a CSV format, we enable integration with existing data analysis tools and workflows, facilitating quantitative analysis without the need for specialized computational skills.

## Results

### LLM-AIx shows high accuracy across various clinical information extraction use cases

To validate the efficacy of our protocol, we conducted experiments across different datasets in different languages and clinical settings. Each use case was designed to test and showcase the protocol’s ability to accurately and efficiently process unstructured text into structured data while addressing specific clinical questions. The performance metrics included accuracy, sensitivity, specificity, F1-score and precision, and the ability to maintain data integrity and privacy. All research procedures were conducted in accordance with the Declaration of Helsinki. Ethics approval was granted by the ethics committee of Technical University Dresden, reference number BO-EK-400092023.

### Fictitious test cases for validation and reuse

Our protocol (Table 1) has demonstrated its efficacy and versatility through application to diverse datasets, notably including the MIMIC dataset^17^ and for psychiatric^16^ and endoscopy report analysis^18^. All of these use cases led to the development of the current pipeline. The pipeline has recently been evaluated for anonymization of real-world clinical letters^19^. Here, we demonstrate the pipeline results for both anonymization and IE of eight fictitious clinical letters for patients with pulmonary embolism, in order to test the pipeline. Llama-3.1 70B correctly identified all patients’ first name and last name, gender, age, date of birth and patient id which led to correct redaction of this information in the reports. The character-wise evaluation results in 99.9% specificity and 100% sensitivity for the anonymization of 8 clinical letters, with 98.2% precision for full name redaction, 94.3% precision for first name and 90% for last name redaction (Table 2). The discrepancy of IE for all personal identifiers (100% precision and sensitivity) and the redaction metrics is because one fictitious patient’s last name matched the provider’s last name (“Miller”, see Supplement) and was therefore also redacted in the exact string match redaction from our pipeline. The fictitious clinical letters report about the clinical course of patients with pulmonary embolism, all of them having different constellations of etiology and risk profiles for this disease. Therefore, we aimed at extracting the presence of leading symptoms such as shortness of breath, chest pain, leg pain or swelling, heart palpitations, cough and dizziness from the clinical letters (Table 3). Additionally, information about the embolism side (left, right or bilateral) should be extracted. All symptoms’ presence was correctly identified with 100% precision and sensitivity, except for the symptom “heart palpitations”, which was missed in one clinical letter.

**Table 1:** Short description of Information Extraction procedure

Step	Name	Timing for TCGA report example
**Stage 1: Problem definition and data preparations**		
1	Define the use case	predefined
2	Assess the input data	predefined
3	Define the validation strategy	predefined
4	Prepare the ground truth data	predefined
5	Download the desired LLMs	~20 min
6	Setup for running the pipeline	~2 min
**Stage 2: Data preprocessing**		
7	Pipeline initiation	~1 min
8	Preprocess the data	~5 min
**Stage 3: LLM-based information extraction**		
9	Prepare the LLM based information extraction	~5 min
10	Run the LLM based Information Extraction	~60 min
**Stage 4: Output evaluation**		
11	A Run evaluation in Information Extraction Mode B Run Evaluation in Anonymizer Mode	~5 min
12	Revise Metrics and Files	~60 min

**Table 2:** Results LLM-Anonymizer (Macro-Averages)

	All labels	Patient name	First name	Last name	Sex	Patient id	Age	Date of birth
Accuracy in %	99.9	99.9	99.9	99.8	100	100	99.9	100
F1	0.994	0.988	0.969	0.944	1.0	1.0	0.958	1.0
Precision in %	98.9	97.7	94.3	90.0	100	100	93.8	100
Recall in %	100	100	100	100	100	100	100	100
FPR	0.0008	0.001	0.0013	0.0026	0.0	0.0	0.0001	0.0
FNR	0.0	0.0	0.0	0.0	0.0	0.0	0.0	0.0

**Table 3:** Results Information Extraction Pulmonary Embolism on Fictitious Reports

	Shortness of breath	Chest pain	Leg pain or swelling	Heart palpitations	Cough	Dizziness	Embolism side
Accuracy in %	100	100	100	88.0	100	100	100
F1	1.0	1.0	1.0	0.8	1.0	1.0	1.0
Precision in %	100	100	100	100	100	100	100
Recall in %	100	100	100	67.0	100	100	100
FPR	0.0	0.0	0.0	0.0	0.0	0.0	0.0
FNR	0.0	0.0	0.0	0.33	0.0	0.0	0.0

### LLM-AIx achieves high accuracy in extracting tumor stage from pathology reports

For demonstrating the capacity of our pipeline in the field of oncology, we additionally used a dataset previously used for IE with *n* = 100 TCGA pathology reports of patients with colorectal cancer, aiming at extracting information about TNM-stage^20^. We ran the experiment with Llama 3.1 70B parameter model and achieved an overall accuracy across all variables extracted of 87%. Extracting the T-stage was accurate in 89% of the reports (F1 Score 0.57, Precision 52%, Recall 68%). The N-stage was accurately extracted in 92% of all reports (F1 Score 0.86, Precision 85%, Recall 87%). The M-stage was accurately extracted in 82% (F1 Score 0.69, Precision 68%, Recall 93%). The number of lymph nodes examined was correctly extracted in 87%. The number of lymph nodes positive for cancer cells was correctly extracted in 90%. Whether the resection margin was tumor free could be identified with an accuracy of 86% (F1 Score 0.92, Precision 87%, Recall 99%, FPR93%, FNR 1%). The extraction of whether lymphatic invasion was present or not achieved an accuracy of 86% (F1 Score 0.82, Precision 70%, Recall 100%, FPR 21%, FNR 0%). We used quantized models because they integrate well with our llama.cpp-based pipeline and require fewer computational resources, and found that the 4-bit quantized Llama 3.1 70B model delivered comparable accuracy to the full-precision version while reducing memory usage from 139 GB to 43 GB in the TCGA dataset example, which makes it more feasible for low-resource environments.

We additionally performed a keyword search for TNM-stages. While keyword-based methods perform well for explicitly stated terms like T-stage but fail with variable or implicit language common in N and M stages, highlighting the superior ability of LLMs to extract clinical concepts from context, as demonstrated by substantially higher performance in identifying stage information without explicit keywords (Supplementary Table 1).

### Main error drivers were conflicting data and OCR failures

A report-by-report error analysis revealed an erroneous ground truth for some reports. When correcting the mistaken ground truth, overall accuracy increased to 88%, 90% for T-stage, 92% for N-stage, 82% for M-stage, 87% for the lymph nodes examined, 90% for cancer-positive lymph nodes, 91% for the tumor free resection margin and 87% for lymphatic invasion (All metrics are shown in Table 4). In some cases, the original report contained conflicting information, e.g., it was described: “resection margin negative (carcinoma less than 1 mm from the radial margin)” which is in fact oftentimes defined as “resection margin positive”, because there is a high risk of tumor recurrence^21–23^. Another example was that “M0 Mx” was supposedly accidentally stated at the same time and “M0” extracted by the LLM. In four cases, the error occurred from wrong OCR recognition of the low-quality scans or handwriting within the report. The LLM identified the right characters, which were already incorrectly stored in the document by OCR (e.g., “N1” was recognized as “N:”, “MA” was recognized as “M1” and the roman numbers “I/IV” for positive lymph nodes were recognized as “1/9” and cited by the LLM as such). We repeated preprocessing of the documents and forced OCR with a different OCR engine, “Surya” and found a slight increase in performance metrics (Table 5). Detailed guides through the experiments with screenshots, also containing prompts and grammar used, are given in the Supplement. These examples demonstrate the successful application of the LLM-AIx-Pipeline for oncological research questions and use cases. It facilitates extracting information from unstructured medical reports with LLMs, thus enabling a structured downstream processing of relevant healthcare data.

**Table 4:** Results after adapting the erroneous ground truth and adjusting the categories

	All	T stage	N stage	M stage	Number of lymphnodes examined	Number of positive lymphnodes	Tumor free resection margin	Lymphatic invasion
Data type		categorical	categorical	categorical	number	number	boolean	boolean
Accuracy in %	88.0	90	92	82	87	90	91	87
F1		0.57	0.86	0.69			0.95	0.85
Precision in %		52	85	68			92	77
Recall in %		68	87	93			99	95
FPR							0.89	0.18
FNR							0.01	0.05

**Table 5:** Results after adapted OCR method (output-categories and boolean)

	All	T stage	N stage	M stage	Number of lymphnodes examined	Number of positive lymphnodes	Tumor free resection margin	Lymphatic invasion
Accuracy in %	89	90	92	88	88	91	91	88
F1		0.6	0.86	0.74			0.95	0.86
Precision in %		58	85	70			93	81
Recall in %		67	87	96			98	93
FPR							0.88	0.15
FNR							0.02	0.07

Standard Prompt: “You are a helpful medical assistant. You are supposed to extract information from a pathology report from a patient with colorectal cancer. I need to know the TNM stage of the patient. This is a system to describe the amount and spread of cancer in a patient’s body, using TNM. T describes the size of the tumor and any spread of cancer into nearby tissue; N describes spread of cancer to nearby lymph nodes; and M describes metastasis (spread of cancer to other parts of the body). If you find no information about the T, N or M stage, give Tx, Nx or Mx, respectively. If there is “pT1” or “pN”, just skip the “p” and give “T1” etc. Additionally, I need information about the number of lymph nodes examined and the number of positive lymph nodes. Let me know if the resection margin was tumor free and if there was lymphatic invasion. If you do not find information about resection margin or lymphatic invasion, say “not mentioned”. This is the report: {report}”.

## Discussion

We present a protocol that enables the extraction of structured information from medical free-text reports using LLMs. This is highly relevant because structured data is essential for enabling more interoperability, efficient analysis, and meaningful clinical and research applications. This need is particularly pressing in oncology, where patient care involves multiple providers, frequent encounters, and an urgent demand for research-driven advancements^24,25^. Traditionally, extracting structured information from unstructured medical text has been challenging due to two major barriers: the lack of labeled datasets required for training conventional NLP models and the labor-intensive nature of the task^26,27^, which is often performed manually. Our pipeline addresses these limitations by offering a solution that allows medical experts to extract structured data without requiring programming skills or prior NLP experience. Unlike other tools such as Apache Tika for OpenWebUI^28^, which primarily parse documents to text and metadata, or Unstract^29^, a general-purpose no-code platform for building document workflows and connecting to vector databases and APIs, LLM-AIx delivers an end-to-end, clinician-oriented pipeline in a single, lean, local browser app. Importantly, domain knowledge remains crucial: Medical experts must define the relevant entities to ensure accurate and meaningful IE.

The protocol relies on LLM inference rather than de novo model training, which balances computational cost. Running the pipeline on consumer hardware is possible, but models with large parameter sizes may require GPUs with substantial VRAM, which could pose challenges in resource-limited settings. In our experiments, moderately sized models (e.g., Llama models with 70 billion parameters in 4- or 5-bit quantization) perform well for oncological use-case IE, thereby facilitating the application within hospital settings.

Despite its advantages, our approach has limitations. Because imperfect accuracy on some variables can affect downstream analyses, our approach is designed to support rather than replace output validation, which enables users to quantify accuracy before using results in statistical modeling and to iteratively refine prompts and benchmark models. High-quality input data is essential, as handwritten or poorly scanned documents may not be processed effectively if the implemented OCR methods are failing. Moreover, OCR techniques themselves require further validation across diverse clinical document types to ensure a reliable textual foundation for LLM-based processing. Additionally, like all LLM-based applications, our method is susceptible to hallucinations, instances where the model generates incorrect or misleading information, and may reflect social biases, such as those related to ethnicity or gender, which can influence output based on superficial cues like patient names^30^. However, careful prompt engineering and hyperparameter adjustments can mitigate this risk^11,31,32^. Errors in real-world clinical documents, such as conflicting or ambiguous information, highlight the need for mechanisms to detect and handle such inconsistencies. Human in the loop approaches can establish a quality control mechanism while still reducing IE time, compared to manual extraction alone, which needs to be proven in further usability research. Although systematic usability testing remains future work, LLM-AIx’s one-command deployment and all-in-browser workflow reduce setup friction. Its UI bundling of IE with per-label metrics and fully local, privacy-preserving operation strongly indicate high usability for clinical workflows. For homogeneous datasets, performance measured on a small, representative test set often approximates performance on the full dataset^18^. Nevertheless, LLMs show additional limitations, which were not specifically evaluated within this work: They present difficulties with basic counting tasks^33^, degraded performance in non-English contexts^34^, and challenges in retrieving specific information from long documents^35^.

The pipeline has demonstrated its versatility in various clinical use cases and can be adapted to any scenario where structured data is needed. Its broader applicability needs further validation, but is already supported by successful validation in other oncology domains, such as lung cancer, as shown by Corso et al.^36^. Unlike traditional NLP models that require task-specific training^37^, LLMs excel in zero-shot settings, making our approach ideal for extracting information across diverse medical documents without additional fine-tuning. For oncology teams, where collaboration between oncologists, radiologists and pathologists is critical for treatment planning, structured data extraction facilitates data sharing across institutions without exposing sensitive text data. It also supports clinical research, where quantitative analysis depends on structured data but is often hindered by the time-consuming manual extraction process. Automating this task could reduce workload and help address personnel shortages in the healthcare sector. In oncology research, structured real-world data is essential for retrospective cohort studies, observational research, and pharmacovigilance efforts^38^. By streamlining IE, our approach has the potential to facilitate large-scale data analysis for studying for example treatment responses, toxicity patterns, and long-term patient outcomes. This is particularly relevant for registries and post-marketing surveillance, where completeness and accuracy of clinical documentation directly impact research validity^39^. Furthermore, the pipeline can assist in maintaining cancer registries, clinical trial documentation, and adverse event monitoring, ensuring that critical patient data is recorded in a structured and accessible format. It also has potential applications in machine learning, where extracted structured data can serve as training labels for predictive models, such as those linking clinical outcomes to imaging data^40–42^. Beyond research applications, structured data extraction can enhance quality assurance and auditing, leading to more complete clinical datasets that support performance evaluations and care quality assessments. Finally, integrating structured information into EHRs allows a more comprehensive and interoperable representation of patient history and treatment, ultimately improving data accessibility for healthcare providers. To support more complex IE use cases and enhance interoperability, the pipeline may require evaluating further prompt engineering and LLM-based fact-checking^43^. Applied in our context, LLMs could assess the factual consistency of extracted elements and reduce the risk of hallucination or misinterpretation by the primary IE language model. Future improvements include translating extracted elements into standardized medical terminologies and ontologies, and adopting interoperable formats such as Health Level Seven International (HL7) Fast Healthcare Interoperability Resources. The framework will also be evaluated in clinical documentation workflows, with expanded output capabilities to enable integration into existing health IT systems.

By showcasing its ease of implementation and relevance in an oncology use case, our approach highlights the immense potential of LLM-based extraction to transform data utilization in oncology research and practice. As the first to present a fully developed, end-to-end solution, we demonstrate not only the extraction of medical information but also the evaluation of the process and the application of different models, setting a foundation to transform data utilization in oncology research and practice.

## Methods

### Overview of the protocol

**Fig. 1:**
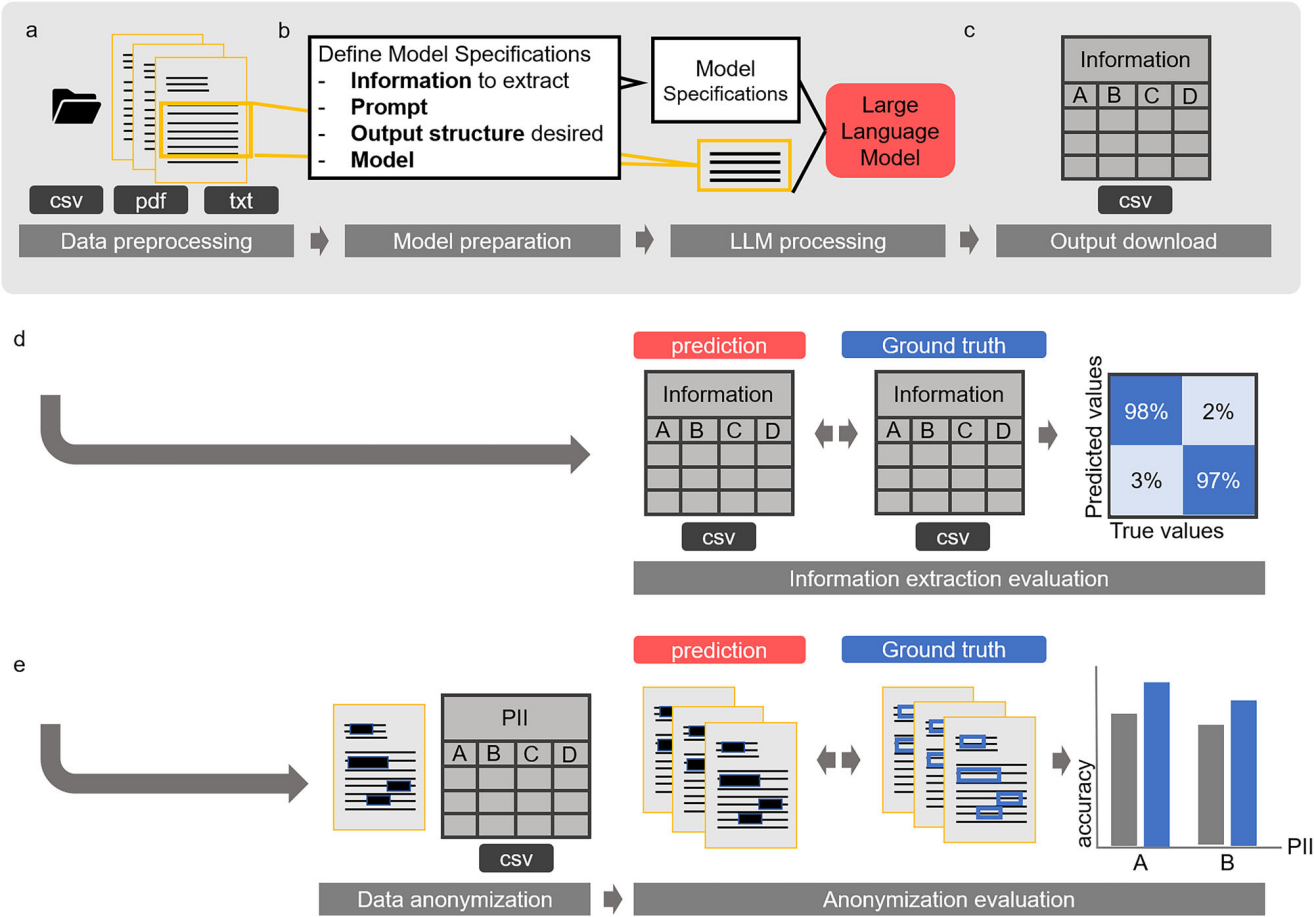
Information extraction workflow. **a** The information extraction pipeline follows a common path that includes data preprocessing, optional Optical Character Recognition (OCR), document splitting, and support for various file formats (CSV, Excel, PDF, or TXT). **b** After preprocessing, users can specify model parameters such as hyperparameters, prompts, and desired output structure. Once these are defined, the LLM-based information extraction process begins. **c** The resulting ZIP file contains the output CSV with LLM predictions of the desired information and the original reports. The evaluation process offers two options. **d** If the pipeline is used for information extraction, it identifies and extracts the required information into a CSV file. This extracted CSV file can then be compared to a ground truth CSV file. Confusion matrices and comprehensive performance metrics are generated to visualize and evaluate the pipeline’s performance. **e** If the pipeline is used for document anonymization, the original documents are redacted to obscure personal identifiers and can be compared to annotated data files. The pipeline automatically generates confusion matrices that visualize matching and mismatching characters, facilitating easy performance evaluation. The anonymization part of this figure is based on the workflow depiction of our previous publication^56^.

The protocol consists of four main stages: (1) Problem definition and data preparation (2) Data preprocessing, (3) LLM-based IE and (4) Output evaluation (Fig. 1). The protocol facilitates any kind of IE from medical free text documents, with a variety of input formats possible. It is easy to use for clinical researchers without Natural Language Processing (NLP) expertise and allows the application of the latest LLMs for medical IE. We have shown that the protocol is broadly applicable to any kind of medical text data. Additionally, our method can be implemented on low hardware resources (e.g., a single graphical processing unit (GPU) with 48 GB video random-access memory (VRAM)), making it more accessible and cost-effective compared to systems with higher computational demands.

### Equipment setup

To set up the pipeline, two main steps are necessary: (1) Model download, (2A) Docker pipeline setup or (2B) Manual pipeline setup:

1) Download the desired models in GGUF format onto your local system.

2A) Edit docker-compose.yml with the correct model path and follow the instructions. Then run the docker image as described in the README.md.

2B) Download the pre-built llama.cpp from Github and follow the installation instructions^44^. Then clone the pipeline’s Github repository. The open-source software for the implementation of the IE experiments is available on GitHub (https://github.com/KatherLab/LLMAIx).

Create a virtual environment and install all necessary python packages within the environment. The detailed implementation of all dependencies and setup is described in the README.md file.

The unstructured text data used to run the pipeline can have different formats. Our pipeline allows processing of PDF, raw text (TXT), CSV, and Excel files. The pipeline can be run fully locally on consumer hardware, such as an NVIDIA RTX 4090 or Apple Silicon M2. For experiments with the Llama 3 70B model, we used a single GPU with 48 GB VRAM (NVIDIA RTX A6000). In theory, the pipeline can also run on GPUs with less VRAM for smaller models: for example, a 12 GB GPU is sufficient for models up to 13B–16B parameters at 4-bit quantization and limited context length. However, running larger models, especially with longer context, requires substantially more VRAM and high-performance hardware. To support local use on resource-limited hardware, we recommend quantized models (4- or 5-bit), which reduce memory requirements while maintaining comparable performance^45^. Nonetheless, even access to 12 GB GPUs can be limited in hospital settings, potentially restricting local deployment to smaller models only.

### Software

The pipeline can be used through a GUI without any programming knowledge. It can be downloaded as a Docker image for a quick setup, including all its dependencies except the model files in GGUF format.

Alternatively, manual setup is possible by installing the required python packages as well as additional software packages (tesseract, llama.cpp) as it is described in the README.md file (https://github.com/KatherLab/LLMAIx). All software packages require a minimum of Python 3.12.

The data preprocessing stage includes different options of Optical Character Recognition (OCR) for processing image-only PDFs. We implemented the popular open source OCR “tesseract” via the package OCRmyPDF, as well as potentially superior alternatives such as “surya”^46^, which can be selected by the User. Default OCR is tesseract.

The protocol adopts the llama.cpp framework which enables the application of a variety of LLMs, formatted in GGUF. It allows LLM inference with state-of-the-art performance on a variety of hardware locally and in the cloud^44^. It is an open-source project that enables the use of Llama models in C++.

### Procedure with step-by-step instructions

#### Define the use case

Preparation for utilizing our protocol involves users to define their specific extraction tasks clearly. This includes specifying the nature of the information to be extracted (for example, identifying complications from endoscopy reports), the format and volume of the data under analysis, and the desired output categories for subsequent analysis. To accommodate documents in various formats (TXT, PDF, or CSV), our protocol standardizes the data into a uniform format (CSV) through automatic conversion and compilation. This standardization is critical for ensuring consistent analysis across diverse datasets.

#### Assess the input data

Identify the raw text data and the format that is available. A patient can have multiple documents, the processing happens per document. If the user plans to process CSV or EXCEL files, the text needs to be filled in a dedicated column, called “report”. Additionally, each text document needs a unique ID in the column “id”. If the user plans to upload single files for each document (e.g., PDF), the files need to be named by the unique ID. Consistency in data labeling needs to be ensured in this data preparation step.

#### Define the validation strategy

This can either be done with document-wide labels (IE-Pipeline) or annotated text data (IE-Anonymizer).

#### Prepare ground truth data

If automated evaluation of the LLM IE pipeline will be performed, a ground truth table of the data or a data subset needs to be provided. If there are unique labels per document, the ground truth needs to be provided in tabular format (CSV or EXCEL), containing all variables that should be extracted as columns and the corresponding ground truth, with the same characteristics as defined for IE. If text annotations and respective labels are supposed to be compared to the LLM output, annotated JSON files (one for each document) need to be zipped. We recommend annotation with INCEpTION^47^, for which the annotation comparison of this pipeline has been optimized.

#### Download the desired LLMs

If not performed beforehands, the user needs to create a folder on the local computer and download the desired LLMs to that folder. All models must be downloaded in GGUF format and can be accessed via huggingface.com. Hugging Face is a company and open-source community that provides a wide range of tools and libraries for NLP and machine learning. We tested several models within the pipeline, among them Llama-3.1^48^ and Llama-3^49^ models, Mistral^50^, Llama-2^51,52^, Gemma^53^ and Phi-models^54^.

#### Setup for running the pipeline

The pipeline can either be run via Docker image or set up and started manually. Detailed descriptions can be found in the README.md. The procedure will be described with the Docker version. Docker needs to be installed^55^.

#### Pipeline initiation

##### A Docker

The docker-compose.yml must be adapted with the correct model path as described in the README.md. Then, run the docker image with “docker-compose up”.

##### B Manually

**Fig. 2:**
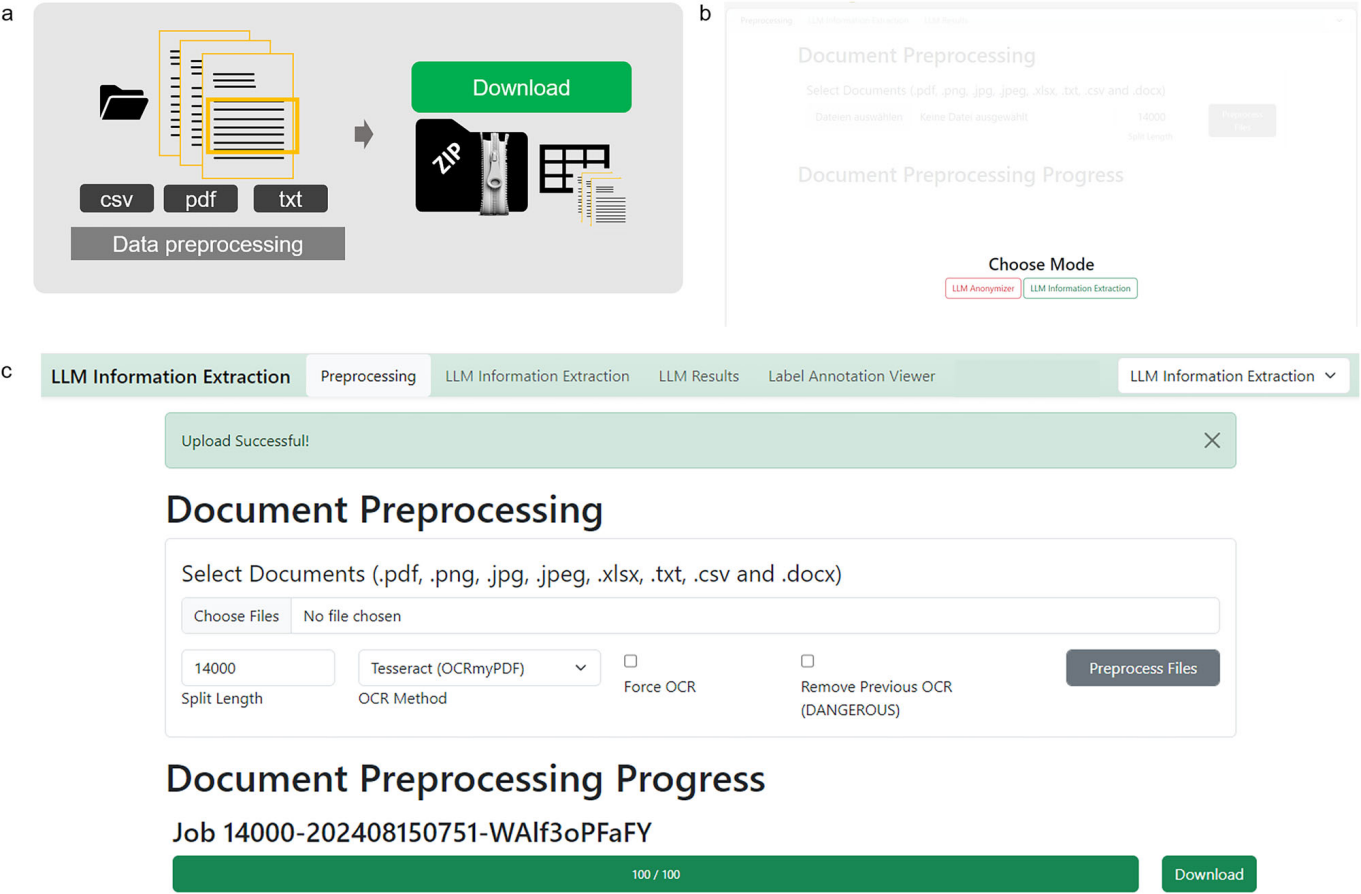
Preprocessing procedure GUI—Stage 2, 7–8. **a** Schematic depiction of the preprocessing stage. Files can be uploaded and will be split to smaller document chunks if necessary according to the split size determined. A zip file containing original documents and a CSV file that organizes all documents for LLM processing can be downloaded in the end **b** When the browser based application is started, the mode of action can be chosen, which is either the “LLM Anonymizer” or the “LLM Information Extraction”. Both modes have the same preprocessing and data processing, however the evaluation part differs. Modes can be switched at any time during the process by adapting the drop-down menu in the upper right corner. **c** The user’s documents can be uploaded in the preprocessing step. Images, as well as portable document format (PDF), raw text, as well as excel or comma separated value (CSV) files are allowed. The split size, which can be determined according to the expected context window size of the LLM, is 14,000 characters per default but can be adapted if necessary. By clicking the “Preprocess Files” button, the preprocessing starts. Progress is indicated with a progress bar. The “Download” button allows you to download and store the preprocessed zip file which will be needed in further processing steps.

To initiate the pipeline, the user activates their virtual environment and navigates into the repository via terminal. There, the app can be started with the terminal command “python app.py –model_path USERMODELPATH”. The application will then be loaded on the local server and can be run browser based. Click on the link provided in the terminal. (Default: https://github.com/KatherLab/LLMAIx/blob/main/app.py). The user chooses the IE-mode based on preferences, either IE or anonymization mode can be chosen (Fig. 2).

### Preprocess the data

After the data has been prepared, it can be preprocessed. The “Preprocessing” tab allows to upload the data files by clicking the “Upload Data” button. PDF files can be uploaded, both “image-only” or PDF documents with a text layer. The name of the PDFs should contain the document id and match the annotated ground truth file id. Additionally, EXCEL or CSV files can be uploaded. They also need to contain an “id” column and a “report” column, containing one report per line. Once all documents are uploaded, the user can select the desired OCR method from a drop-down menu and specify the number of characters. If needed, the document will then be split to accommodate the limited context windows of certain models. After clicking the “Preprocess Files” button, a progress bar appears and indicates the preprocessing status. As soon as finished, the preprocessed data needs to be downloaded as a zipped folder. This zip folder contains all original documents in a separate PDF file as well as a CSV file. (Fig. 2).

**Fig. 3:**
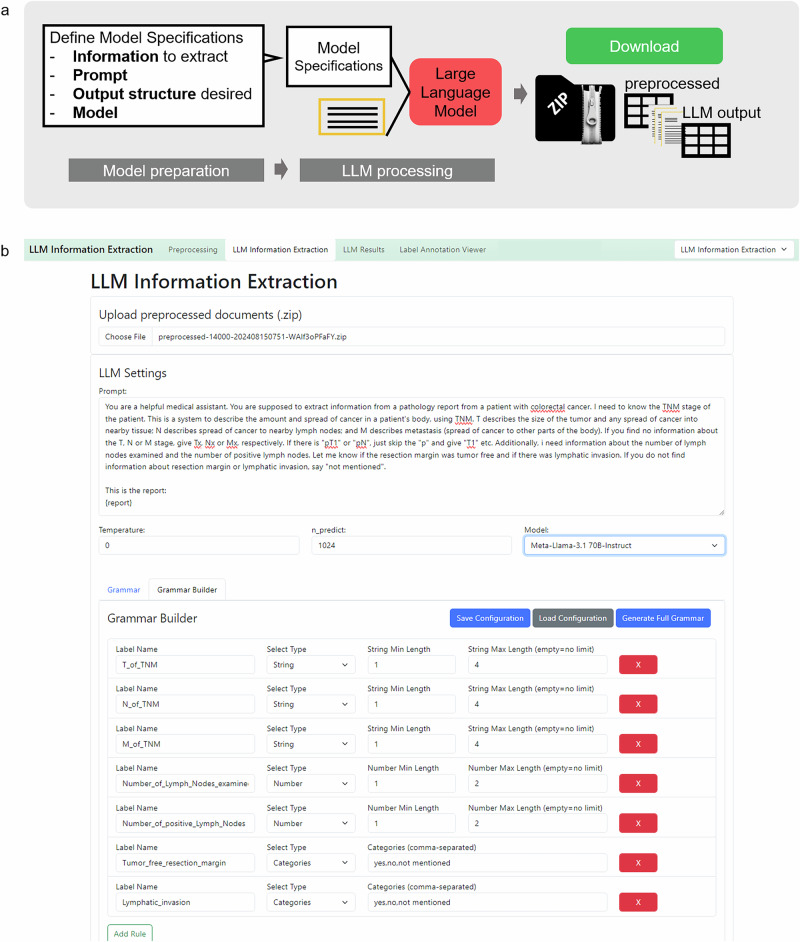
LLM-processing procedure—Stage 2, 7–8. **a** Schematic depiction of the LLM-processing stage. The large language model (LLM) based information extraction requires uploading of the preprocessed zip file. Then LLM Settings can be determined. The prompt field allows inserting a specific prompt. At the “{report}” indicator, your original report text will be inserted. Additionally, LLM hyperparameters can be set (Temperature and tokens to be predicted (n_predict)) and the desired model can be chosen via a dropdown menu. To ensure a consistent output structure, the model can be given a JavaScript Object Notation (JSON) schema. This can either be defined manually, which is prone to errors. Therefore, we implemented a “Grammar Builder” that allows to define your feature name and values to be extracted. The grammar configuration shown in (**b**) can be downloaded and stored on your local computer and loaded whenever needed. With the button “Generate Full Grammar”, it will be loaded to the processing mask. Afterwards, “Run LLM Processing” starts the information extraction. The model chosen will then be loaded on the local Graphical Processing Unit (GPU), before the information extraction starts. This is indicated by a loading circle in the Graphical User Interface (GUI) or the uploading dot indicators in the terminal. As soon as the model is successfully loaded to the GPU, the LLM-based processing starts and progress is indicated with a progress bar.

### Prepare the LLM based information extraction (Fig. 3)

#### Model selection

The user specifies the desired model by choosing it from the drop-down menu. We have predefined the most common open source models. Additional models can be added by downloading them to the predefined model folder and adding them to the “yaml file”.

#### Upload the preprocessed zip file

With the “upload” button, the preprocessed zip file can be uploaded for further processing.

#### Prompt definition

The prompt can be defined in the “Prompt” field and can be customized according to the user’s needs. At the location of “report” in parenthesis, the respective report will be inserted, therefore this element needs to stay within the prompt as is. The location can be varied upon necessity. We recommend a prompt that is structured in two main parts: Giving background to the model and instructing the model with the task. If the IE-task is more complex, it can be helpful to insert examples for few-shot prompting that leverages in-context learning of the models. It is important to demonstrate examples in the same format as the output is desired.

#### Grammar specification

The section “Grammar” contains a JSON schema defining the output structure of the model. The grammar-based sampling approach that is applied here enables the user to flexibly define the features to be extracted from the text and their possible outcomes. The desired model output JSON schema can either be adapted manually (error-prone and therefore not recommended) or with the “Grammar Builder”. The user can assign a label name for the information to be extracted and select the desired output format (string, boolean, categories, number) and further specify the categories (in a comma-separated list) and the character length of the string and number. Once defined, the grammar can be downloaded and stored as a CSV file by clicking the “Save Configuration” button. Whenever the same configuration is required again, it can be uploaded via the “Load Configuration” button. Once the grammar is set up correctly, the user loads it into the “Grammar” section with the button “Generate Full Grammar”.

If prompt, grammar and hyperparameters are correctly defined and the preprocessed file is uploaded, clicking the button “Run LLM Processing” initiates the process.

#### Run the LLM based information extraction

First, the respective model will be uploaded onto the local server. This might take a few seconds up to several minutes, depending on the model size and hardware specifications. When the model is loaded, processing starts and a progress bar indicates the remaining amount of reports and time for the process. The remaining time is corrected after each report is processed. After the process is finished, the user can download and store the processed zip file. This file contains all original reports as PDFs, the preprocessed CSV as well as an output CSV that contains all LLM answers as well as meta-information including prompt and hyperparameter settings.

## Run evaluations

### A run evaluation in information extraction mode

**Fig. 4:**
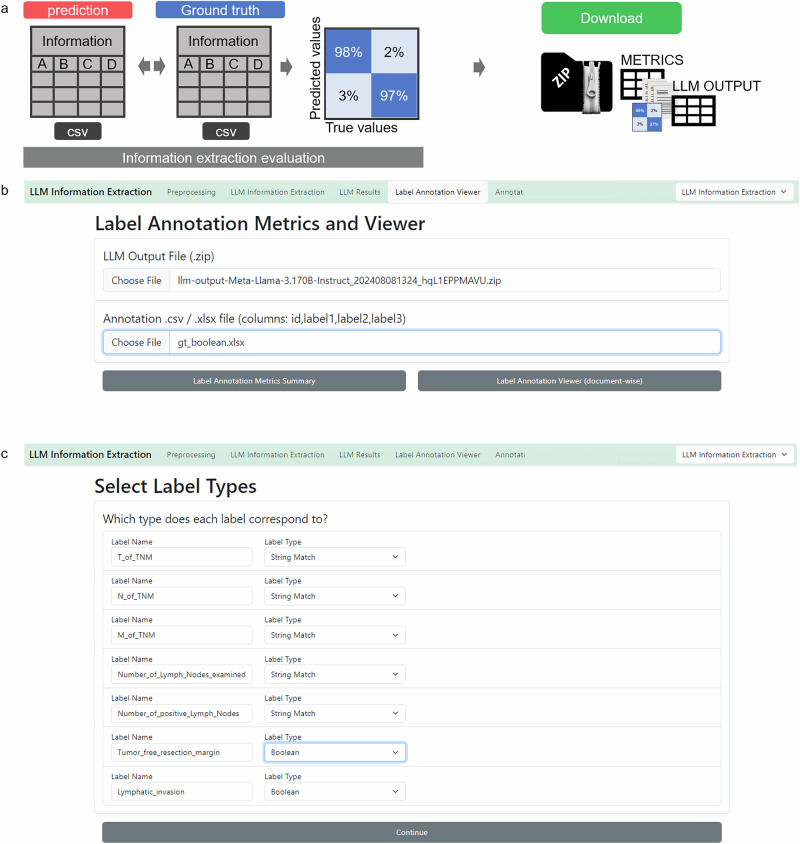
Information extraction evaluation initiation—Stage 4, 11A. **a** Schematic depiction of the evaluation process. LLM output and human made ground truth can be compared automatically and metrics as well as confusion matrices will be calculated and displayed. Those can be downloaded in a metrics-zip file which contains metrics, figures, original documents and LLM output. **b** The LLM Annotation Metrics and Viewer requires upload of the LLM output file (zip-file) and the annotated file (CSV or EXCEL). After initiating the comparison of output file and ground truth file with the “Label Annotation Metrics Summary” button, the label data types of the extracted information variables need to be confirmed (**c**).

The “Label Annotation Viewer” enables the uploading of both the output ZIP file and the ground truth CSV file. It is essential to ensure that the IDs and variable names are unique and matching in both files (Fig. 4a). The process can be initiated by clicking “Label Annotation Metrics Summary” to obtain all metrics and “Label Annotation Viewer” to review results on a document-by-document basis. Before results are obtained, data types of the extracted variables have to be confirmed to ensure proper evaluation (Fig. 4b).

### B run evaluation in anonymizer mode

To evaluate the output in the Anonymizer mode, the output zip file can equally be uploaded. The ground truth needs to contain annotations for the individual reports in JSON format. For annotation tasks, we used the open source annotation tool “INCEpTION”^47^. The annotated JSON exports need to be zipped and can then be uploaded equally to the IE evaluation.

### Revise metrics and files

**Fig. 5:**
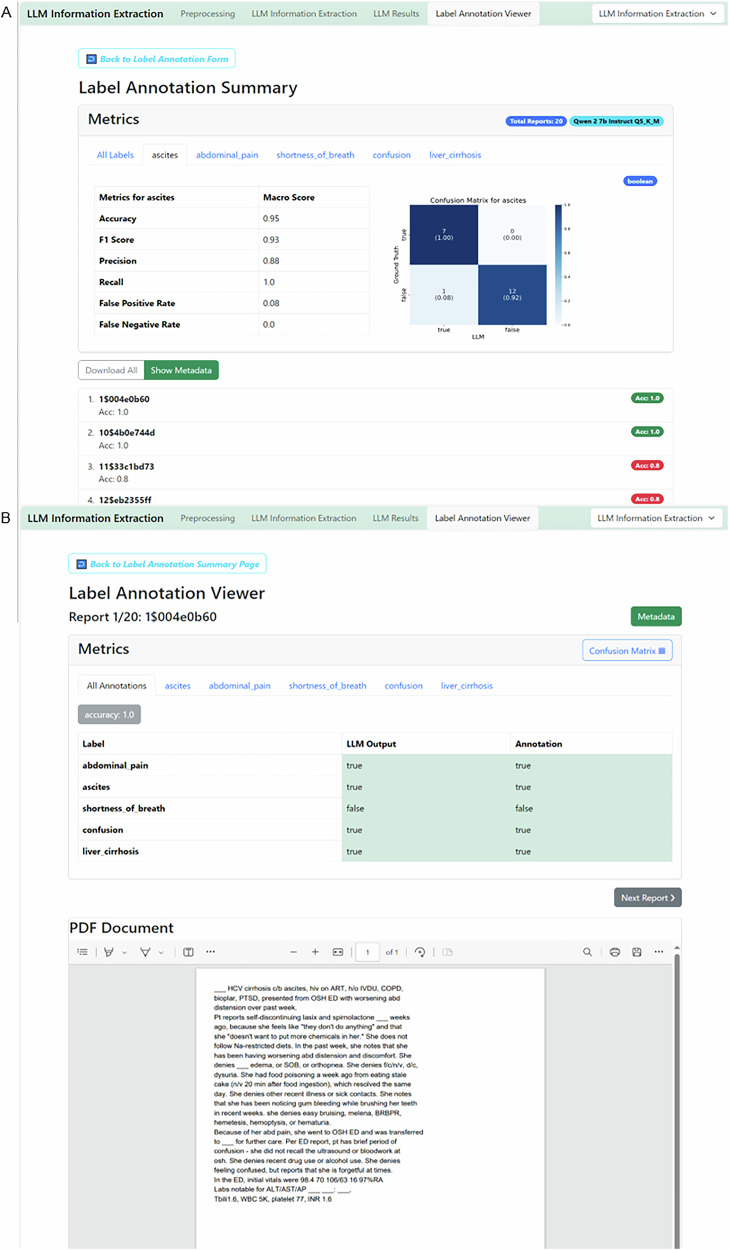
Evaluation of information extraction results GUI, stage 4, 12. **A** The ”Label Annotation Summary” displays overall metrics and metrics per label. **B** Clicking on the respective report displays the original text and the model output as well as respective ground truth provided. This enables a seamless evaluation and document revision process.

The “Label Annotation Summary” provides global metrics for the experiment by comparing the output data to the ground truth data. For boolean IE, it presents a comprehensive set of metrics, including Accuracy, F1 Score, Precision, Recall, False Positive Rate, and False Negative Rate, both across all reports and variables, and for each variable individually. A confusion matrix visualizes true and false positives and negatives (Fig. 5A). For categorical variables, the confusion matrix shows matches between ground truth categorical values and output values. For string variables, a string match is displayed. In anonymization tasks, a character-wise overview of truly and falsely redacted characters is given, both as a global metric and for each patient identifier individually. Additionally, each report is listed below the metrics overview. Users can individually select and review each report to double-check and compare the LLM output with the original text and ground truth annotation (Fig. 5B).

## Supplementary information

Supplementary Information

## Data Availability

All fictitious data is available in the example section of the GitHub repository (https://github.com/KatherLab/LLMAIx). The results for the real world pathology dataset shown here are in whole or part based upon data generated by the TCGA Research Network: https://www.cancer.gov/tcga.

## Code availability

The open-source software for the implementation of the IE experiments is available on GitHub (https://github.com/KatherLab/LLMAIx).
