# Supplementary material for: A software pipeline for medical information extraction with large language models, open source and suitable for oncology
Source: NPJ Precis Oncol. 2025 Sep 17;9:313. doi: 10.1038/s41698-025-01103-4 (PMC12443949; doi:10.1038/s41698-025-01103-4)
Supplement: Supplementary file 1 — Supplementary Information [file 41698_2025_1103_MOESM1_ESM.docx]

# Supplement

## Comparison with other methods

Until now, due to the shortcomings of other methods, the gold standard in medical IE is labor intensive, manual IE by medical documentaries or medical staff. Traditional methods such as machine learning named entity recognition (NER) methods typically require the extraction of fixed entities and offer limited flexibility. To name an example, they extract all names, dates and locations from a text, without being able to interpret the context. If only the surgery date was needed from a report, this could not easily be identified among all dates mentioned within a text.

In contrast, our LLM-based approach allows for the flexible definition of entities to be extracted through advanced prompt engineering and in-context learning capabilities. This adaptability makes it more suitable for the dynamic and varied needs of medical data analysis.

**NLP in the pre-transformer era - Beginnings of pre-trained models**

Initially, IE relied on hand-crafted rules and required extensive manual efforts to define patterns which were limited in their adaptability to different domains ^57,58^. Machine learning techniques, which used labeled data to train models, improved the IE performance in the NLP domain ^59^. These methods leveraged features as part-of-speech tags; such as nouns, verb or adjectives ^60^, syntactic structures; such as noun phrases, verb phrases and adjective phrases ^61^, and lexical cues, which are words indicating a specific relation and entity, such as intensifiers like “very” in the sentence “she was very happy” ^62^, to improve the accuracy of entity recognition and relation extraction. Notable algorithms like Hidden Markov Models (HMMs) and Conditional Random Fields (CRFs) were widely adopted for these tasks ^63,64^. Non-neural methods such as n-gram models ^65–67^, and neural network methods, particularly recurrent neural networks (RNNs) with long short-term memory (LSTM) and convolutional neural networks (CNNs) were able to improve capturing contextual information ^68^. However, labeled data is scarce, particularly in the medical domain, and as a result, unsupervised and semi-supervised learning approaches were advanced simultaneously. They aim to utilize large amounts of unlabeled text to automatically discover patterns in the data. Word embeddings, which are representations of words in continuous vector spaces, such as Word2Vec or GloVe, further enhanced the generalizability across different contexts ^69^. ULMFit followed as one of the first approaches for pre-trained models ^70^. However, all of these methods suffered from limited context understanding in document level, do not capture polysemy, have a fixed vocabulary size, require large text corpora for training and were so far insufficient for IE in the medical field ^71^.

**New prospects with LLMs**

The development of the transformer architecture, a deep learning architecture that is based on multi-head attention ^72,73^, substantially changed the NLP landscape: Especially the introduction of Bidirectional Encoder Representations from Transformers (BERT) and Generative Pre-trained Transformer (GPT) advanced the field. These models capture language patterns and context, and subsequent fine-tuning of pre-trained models on specific tasks achieved good results in entity recognition ^74^. BERT-based models have also been established for the biomedical domain (BioBERT, SciBERT, ClinicalBERT, BioMedRoBERTa) and tested on several benchmark datasets (GLUE, MuliNLI, SQuAD) ^75,76^. Nevertheless, BERT models require fine-tuning for successful IE, which requires procedure and programming knowledge and have very limited context length ^10^.

LLMs, which have larger parameter sizes than BERT-based language models, have shown great potential in classical IE tasks ^77^. They offer a high zero-shot performance and shift the task solving field towards immediate prompt engineering instead of fine-tuning and model training. In-context-learning, which does not alter the model's weights and has the advantage of using purely natural language, potentially allows medical staff without programming knowledge to seamlessly integrate these tools into their daily routines. Furthermore, it provides maximum flexibility to extract contextually relevant information as specified by the requester, requiring minimal programming knowledge, making it ideal for the information extraction process in the medical field ^78^. The strength of our approach lies in its robust performance across datasets of any size, ensuring efficiency and accuracy whether analyzing a single report or aggregating insights from a vast collection of documents.

**Supplementary Table 1 - Keyword search.** A simple keyword search for possible options of the TNM stage categories yielded limited results, especially for the not-explicitly stated N-stage and M-stage.

| TNM stage | Keywords | Exact matches | More than one keyword found | Wrong keyword found | No keyword found |
| --- | --- | --- | --- | --- | --- |
| T | T0,T1,T2,T2a,T2b,T2c,T3,T3a,T3b,T3c,T4,T4a,T4b,Tx | 83 | 10 | 2 | 15 |
| N | N0,N1a,N1,N1b,N2,N2b, N2a,Nx | 54 | 13 | 3 | 43 |
| M | M0,M1,Mx | 13 | 0 | 2 | 85 |

## Troubleshooting

| Step | Problem | Possible Reason | Solution |
| --- | --- | --- | --- |
| Data preprocessing | | | |
| 6 | Expecting xx lines in, saw yy | Erroneous CSV, wrong encoding | Make sure you store the data in proper CSV files and ensure UTF-8 coding. |
| LLM-based information extraction | | | |
| 10 | Model could not be loaded on Server | GPU capacity exceeded, too many processes are running in parallel or model is too big for hardware resources | Terminate other processes running on the GPU. If model size is the problem, use a smaller model or higher quantization. |
| 10 | LLM processing very slow | Pipeline is run on CPU of consumer hardware | Loading the LLM to a gPU, if available, increases the speed of the process. Default model upload in the pipeline is GPU upload. |
| 10 | Empty LLM output | LLM output is invalid JSON because the model did not answer anything, did respond with invalid json or the answer was cut off because n_predict is too low. | Increase n_predict. Test with less complex grammar, prompt and other models. |
| 10 | Empty LLM output | N-predict might be too low | Increase n_predict. Test with less complex grammar, prompt and other models. |
| Output Evaluation | | | |
| 11 | Mismatch LLM output with annotation | LLM hallucinated  **Supplementary Figure 11** | Improve explanation for this variable in prompt |
| 11 | Mismatch LLM output with annotation | Ground truth is wrong | Refine the ground truth through expert discussion |
| 11 | Mismatch LLM output with annotation | Input text data is conflicting. **Supplementary Figure 12, 13** | Refine the ground truth through expert discussion |
| 11 | Mismatch LLM output with annotation | Annotation is coarser than LLM  **Supplementary Figure 14** | Refine the ground truth through expert discussion |
| 11 | Mismatch LLM output with annotation | Annotation is more detailed than than LLM  **Supplementary Figure 15** | Refine the ground truth through expert discussion |
| 11 | Mismatch LLM output with annotation | Classes are not sufficiently distinguishable, e.g. model is unable to distinguish between “no” and “none”.  **Supplementary Figure 16** | Classes need to be defined clearly mutually exclusive and commonly exhaustive and close to interpretation of natural language. E.g. If the presence of “lymphatic invasion” is questioned, the answer options are either “yes”, “no” or “not mentioned”. These classes are closer to human language and therefore clearer for an LLM than “yes, no, none”. |
| 11 | Mismatch LLM output with annotation | Wrong OCR due to bad quality PDFs.  **Supplementary Figure 17** | If documents contain a high share of hand-written information and PDFs are of very bad quality, text cannot be extracted correctly, the extraction failure therefore arises from data preprocessing and can be overcome by selecting another OCR method for preprocessing. “Surya” surpasses “tesseract” in general and the visionLLM Phi or trOCR are better in detection hand written text. |
| 11 | Mismatch LLM output with annotation | The LLM output is accurate but has minor character discrepancies compared to the annotation.  **Supplementary Figure 18** | Desired output needs to be specified as detailed as possible within the prompt and grammar. |
| 11 | Mismatch LLM output with annotation | Information is present but could not be detected by the LLM.  **Supplementary Figure 19** | Add a more detailed explanation within the prompt and give few-shot examples. |
| 11 | Mismatch LLM output with annotation | LLM extracts information better than human rater.  **Supplementary Figure 20** | Refine the ground truth through expert discussion |
| 11 | Mismatch LLM output with annotation | LLM lacks implicit knowledge. **Supplementary Figure 21** | Desired output and definition needs to be specified as detailed as possible within the prompt. Add few-shot examples. |
| 11 | Mismatch LLM output with annotation | Output categories are misdefined. **Supplementary Figure 22** | Make sure the output categories are the same as in annotated ground truth |

## Timing

The time required to complete this protocol varies depending on the size of the dataset, the LLM’s size and the available computational resources. The most time-intensive steps are Step 9 “***Prepare the LLM based information extraction”,*** 10 “***Run the LLM based information extraction”*** and Step 12”***Revise Metrics and Files***”. The time estimates provided in **Table 1** are based on running the protocol on the example dataset (comprising n=100 TCGA pathology reports with Llama 3.1 70B model) using a NVIDIA RTX A6000 GPU on a Windows workstation.

## Guide through anonymization example


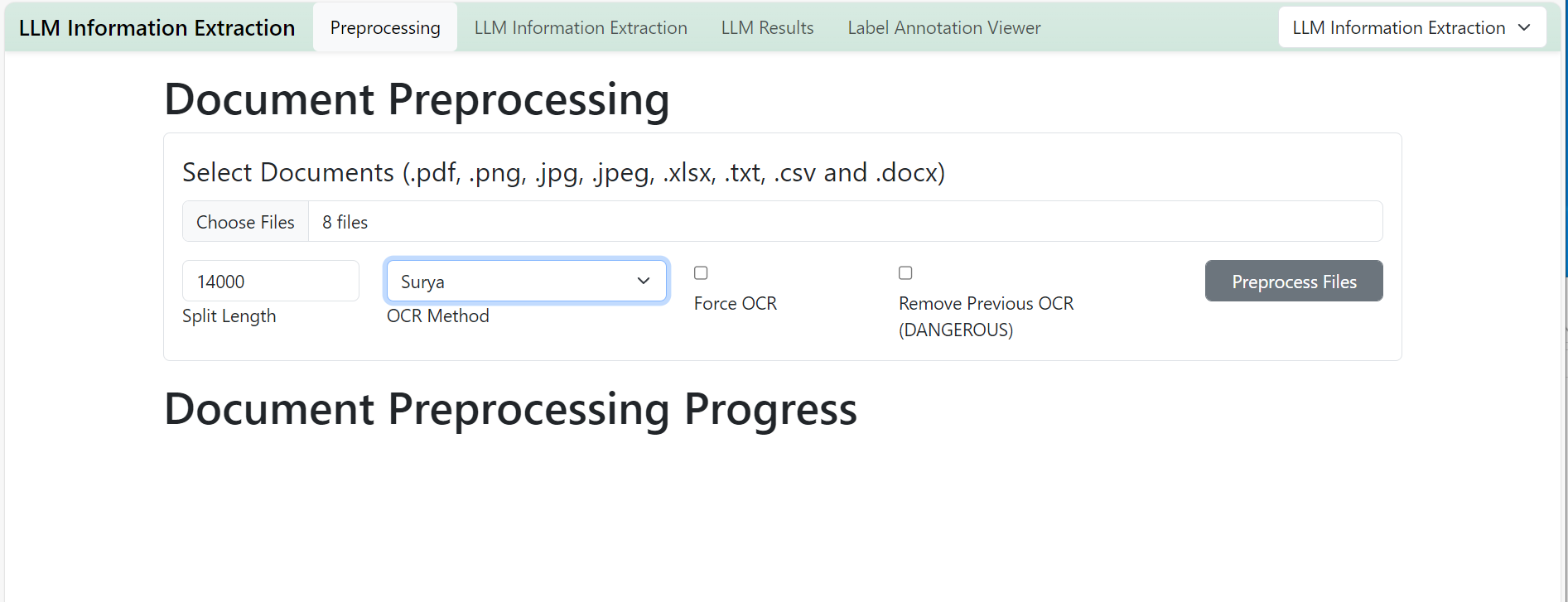


### **Supplementary Figure 1**

Preprocessing of fictitious clinical letters. Preprocessing is performed equally in the LLM Information Extraction mode and in the Anonymizer mode. The 8 fictitious letters were uploaded and the Optical Character Recognition (OCR) method defined. Since all PDFs already contained text, no OCR was necessary. The preprocessing process can be started by clicking the “Preprocess Files” button. A progress bar indicates the process and a “Download” button allows downloading the preprocessed files as a zip file.


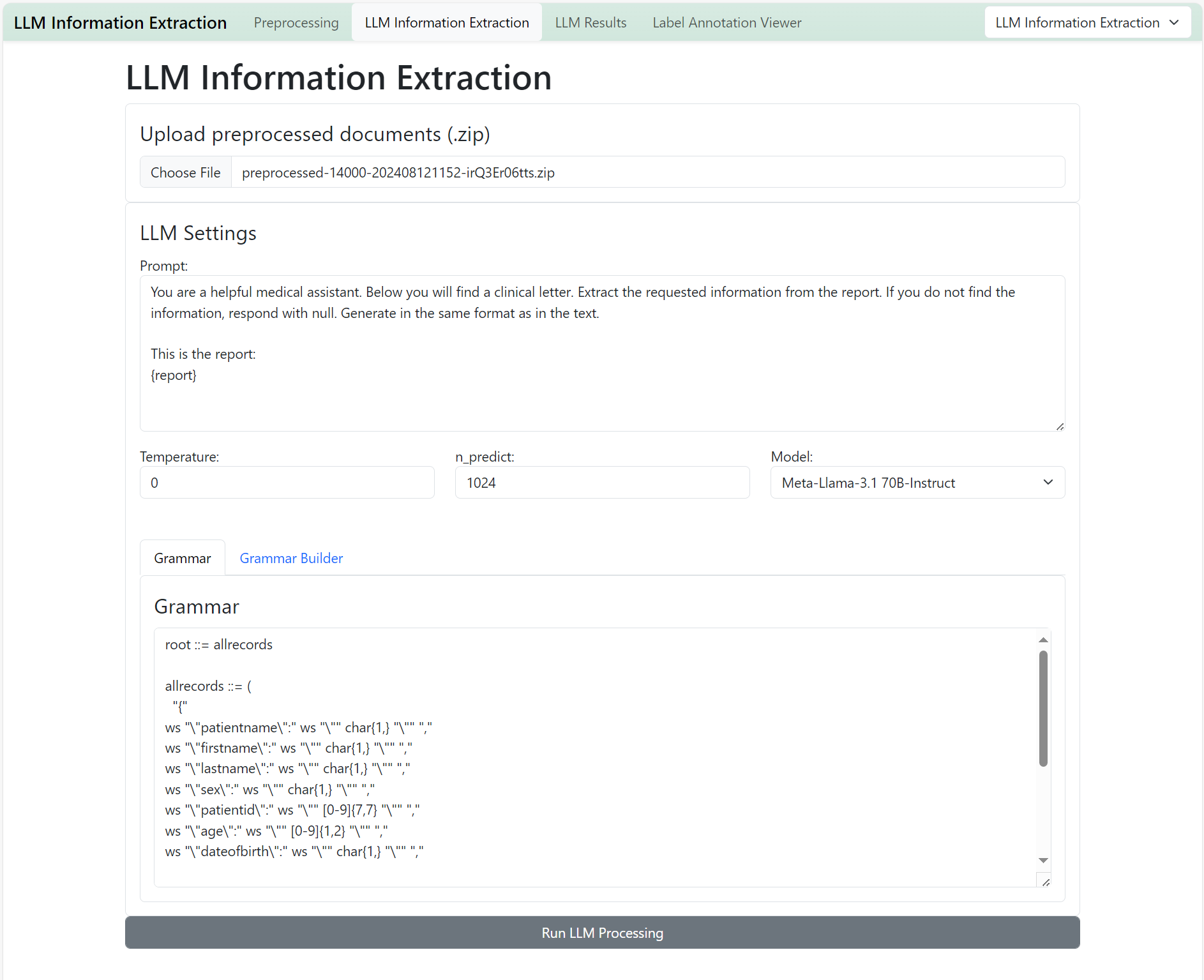


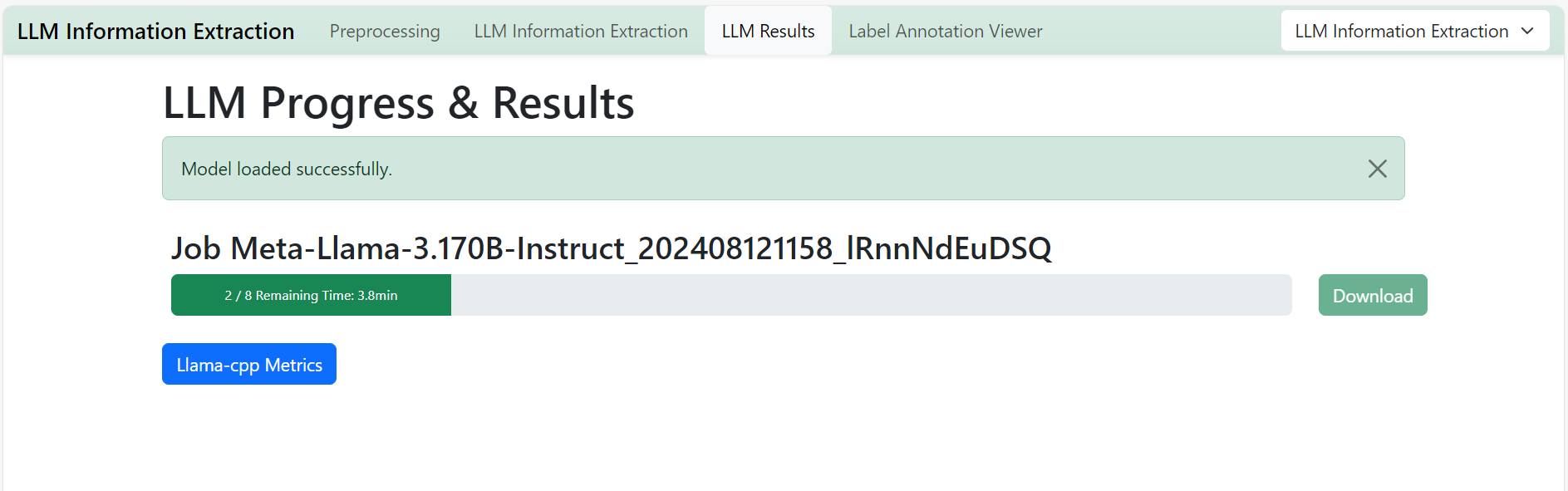


### **Supplementary Figure 2**

Preparation of LLM based information extraction. **A** The preprocessed zip file was uploaded in the respective field (“Upload preprocessed documents”). Within the LLM settings, the prompt can be adapted. The prompt used for the example anonymization task is given here. The hyperparameter temperature was set to zero to ensure the most deterministic LLM output and the model chosen was Meta’s Llama 3.1 70B in 4-bit quantization and GGUF format available from “huggingface”. With the grammar builder, the shown grammar could be defined to ensure consistent JSON formatted output with the desired variables. The button “Run LLM Processing” allows to start the LLM based information extraction. **B** The correct model loading is indicated with a green bar stating “Model loaded successfully”. A progress bar displays the remaining process time and a “Download” button allows downloading the output zip file as soon as the process is finished. This process can be performed in both Information extraction and Anonymizer mode.


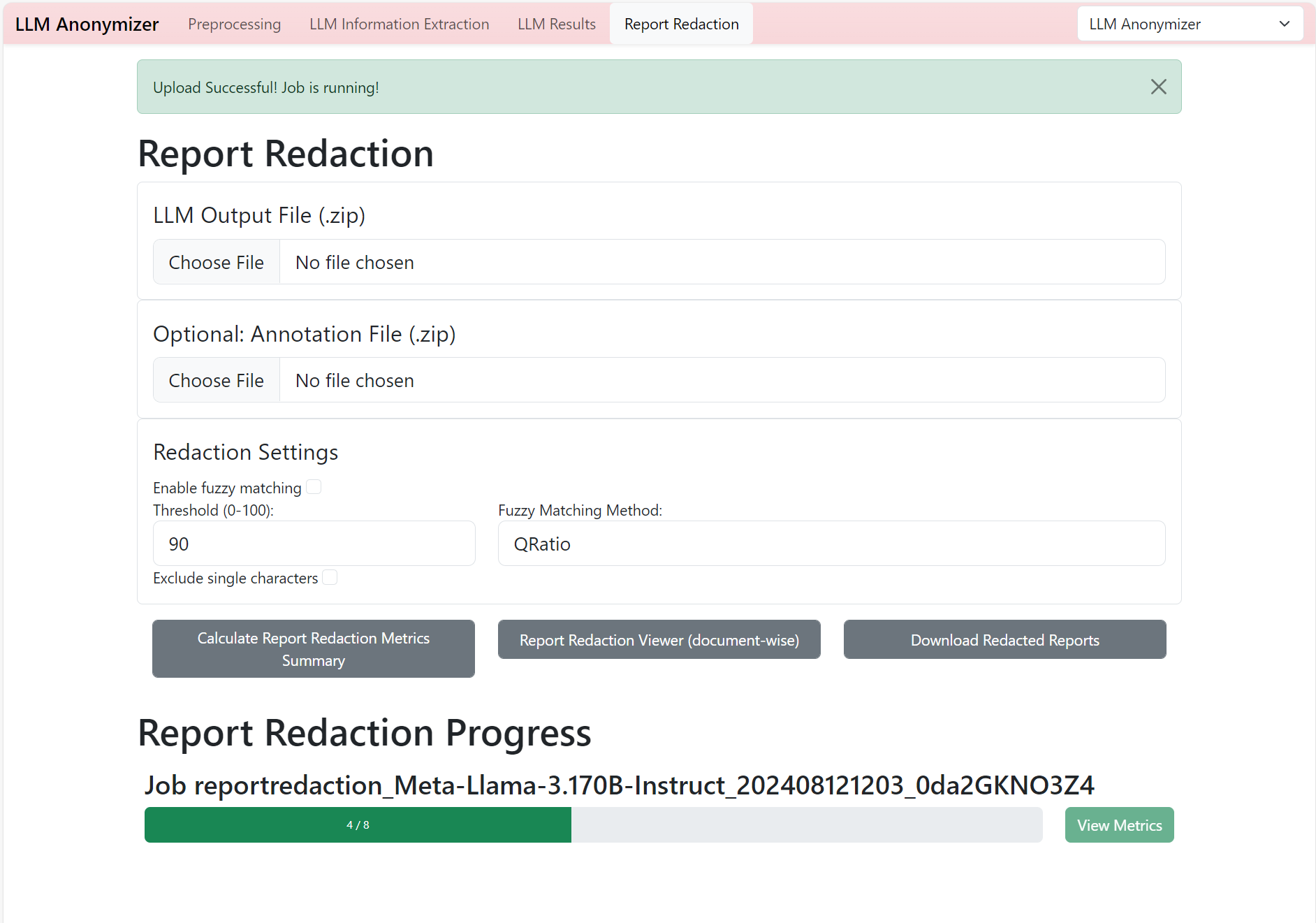


### **Supplementary Figure 3**

Report Redaction. The analysis and report redaction part is specific to the Anonymizer mode, therefore the mode needs to be switched in the right upper corner of the window. The LLM output zip file was downloaded for the 8 fictitious clinical letters as well as the annotation file (human annotations were performed with the annotation tool “Inception” and downloaded as JSON files. These JSON files were zipped and uploaded). The extracted identifiers were now redacted within the original clinical letters with exact character matching. The pipeline additionally allows for “fuzzy matching”, a technique used to find strings that are approximately rather than exactly equal. This can be useful for typographical errors or variations in spelling. Two options are available: Qratio and Wratio. Qratio compares two strings and provides a similarity score between 0 and 100, zero meaning that the two strings are completely different. Wratio adjusts the basic Qratio by string length and variations. The threshold can be flexibly set based on the user’s needs between 0 (everything matches, regardless of similarity) and 100 (only exact string matches are considered).


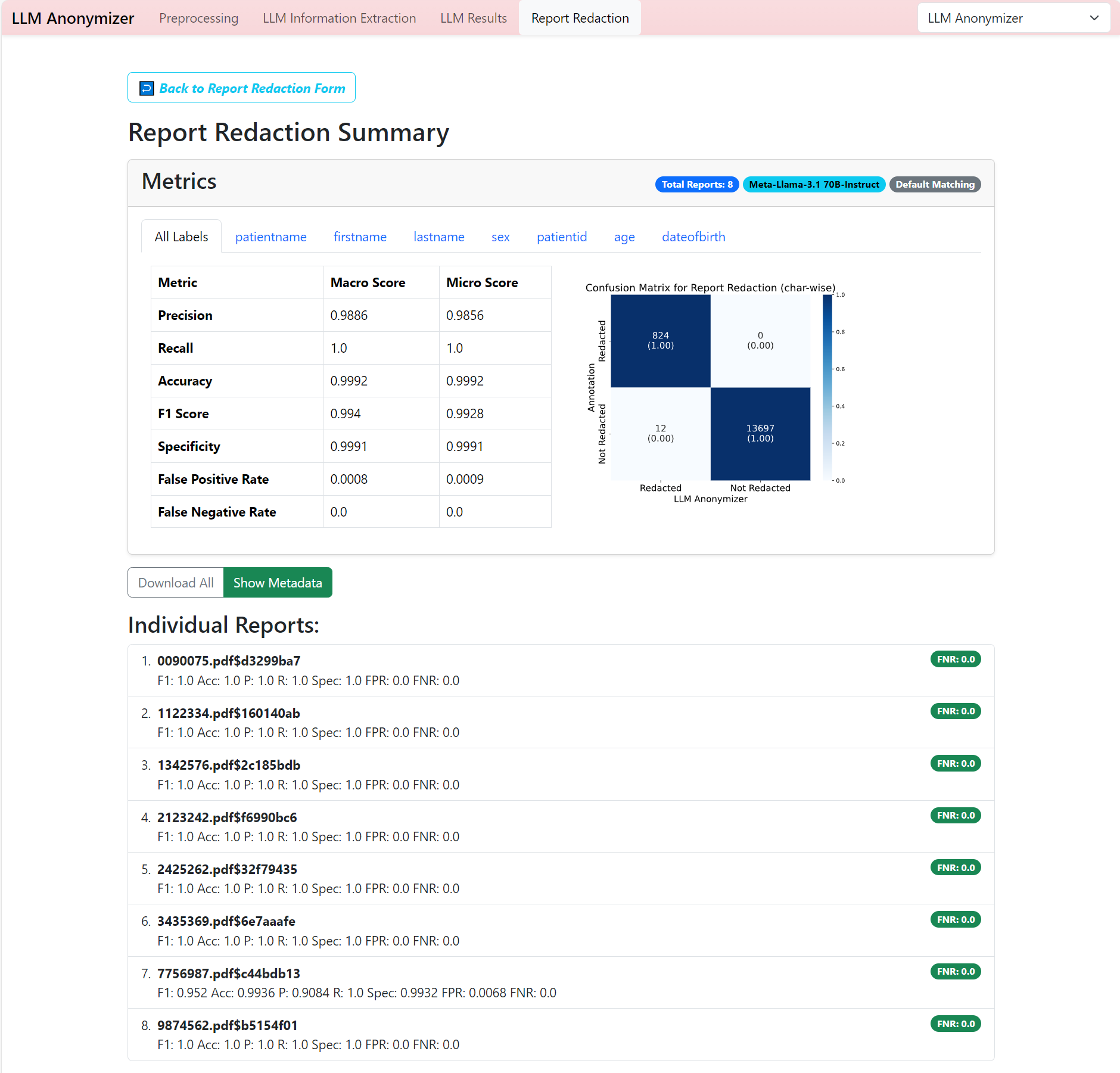


### **Supplementary Figure 4**

Report Redaction Summary. The report redaction summary displays character-wise matching metrics between the human annotations and the pipeline-redacted documents. Macro and Micro scores are shown for all labels as well as for each variable extracted. Blue and grey tags indicate the total number of reports processed (8 fictitious clinical letters), the model used (Meta-Llama 3.1 70B) and the matching algorithm (Default: Exact match). Additionally, false negative rates (FNR) are displayed for all clinical letters individually. These documents can be opened and reviewed by clicking on each document in the list.


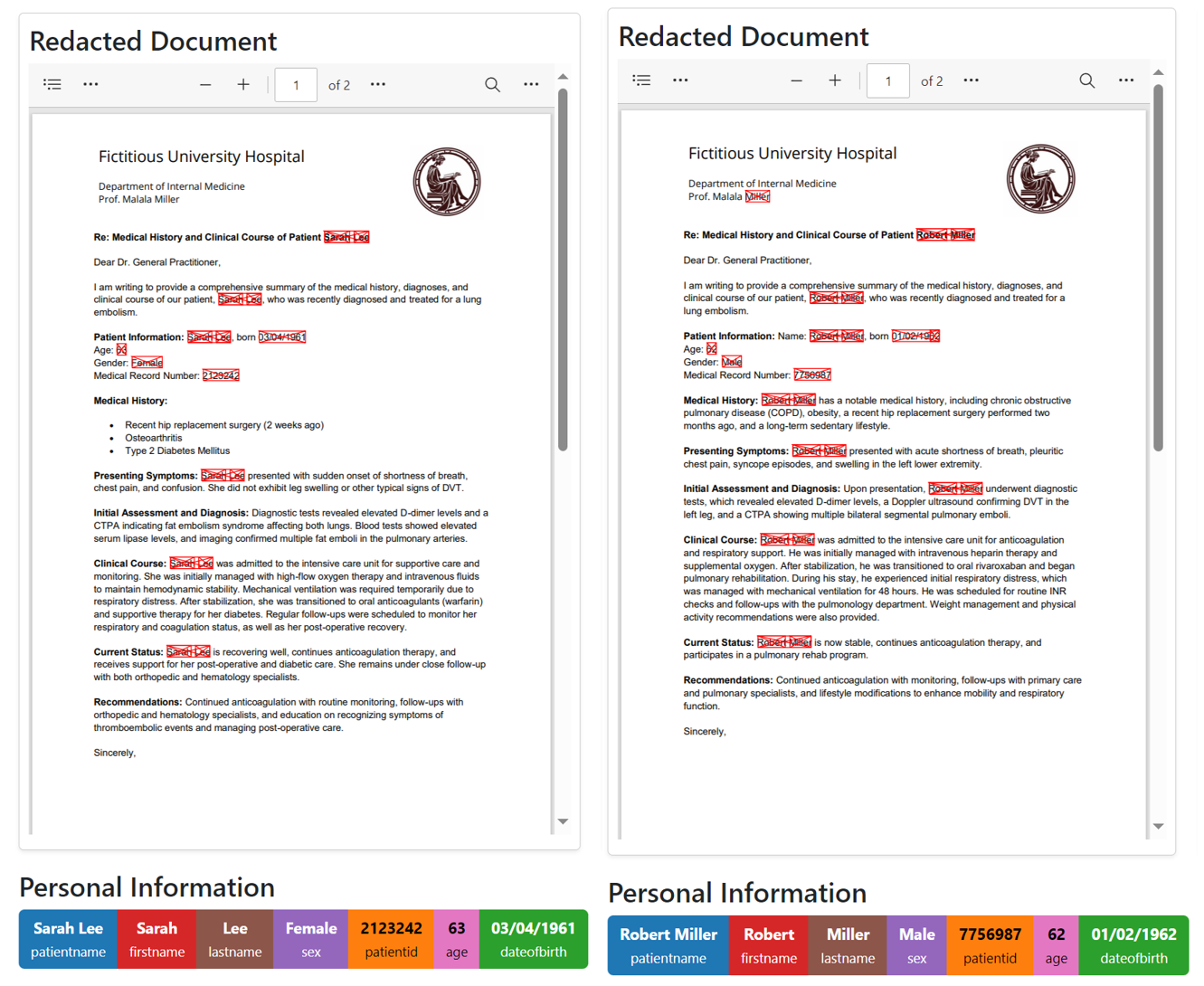


### **Supplementary Figure 5**

Redacted document view. Each report can be reviewed individually. Two examples from Anonymization example with 8 fictitious clinical letters are shown here. The “Personal Information” bar displays the extracted personal information. The original clinical letter can be reviewed and red boxes indicate The extracted information is then displayed separately and red boxes indicate the fields that are blackened when downloading the redacted documents.

### Fictitious Example Reports
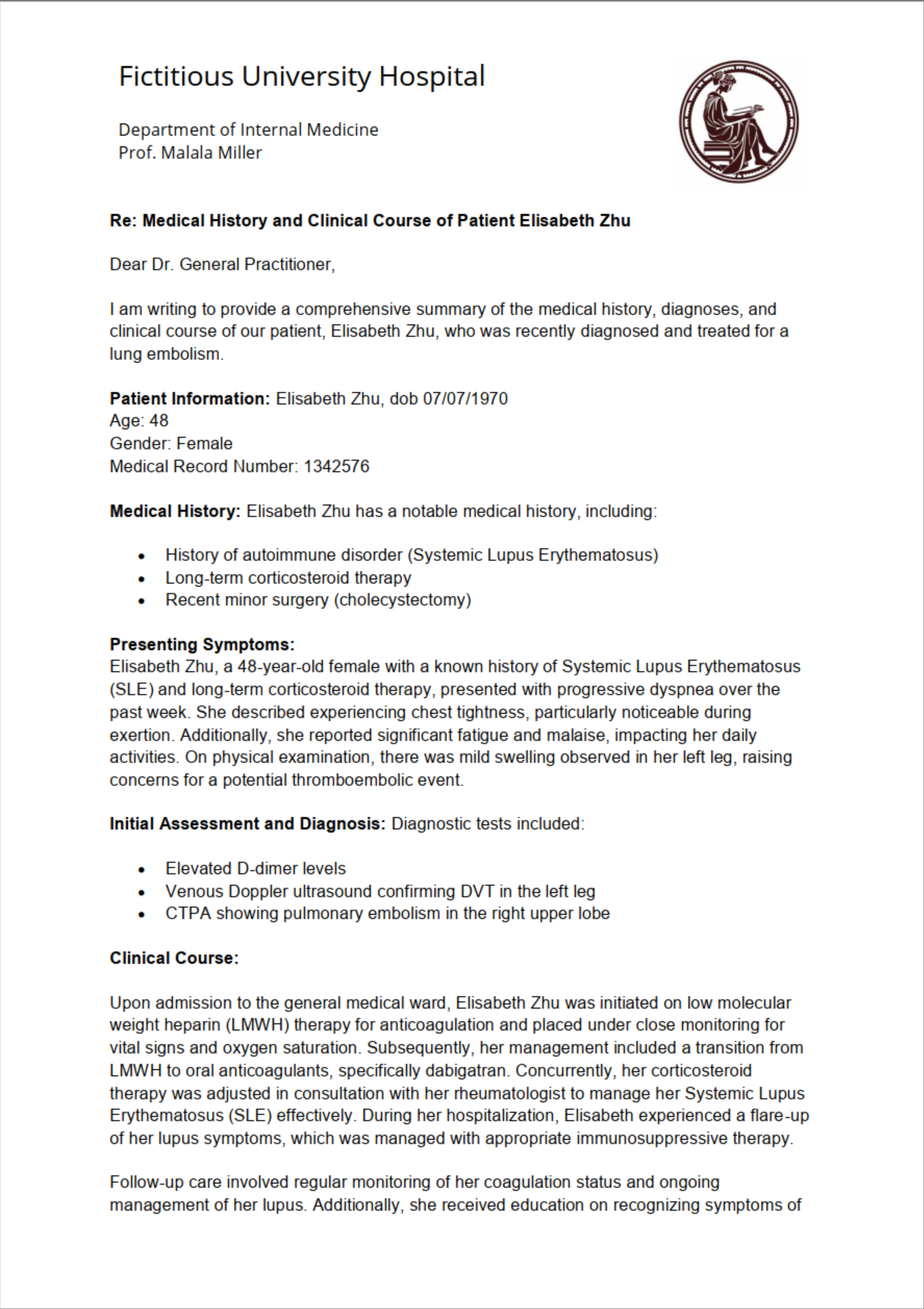

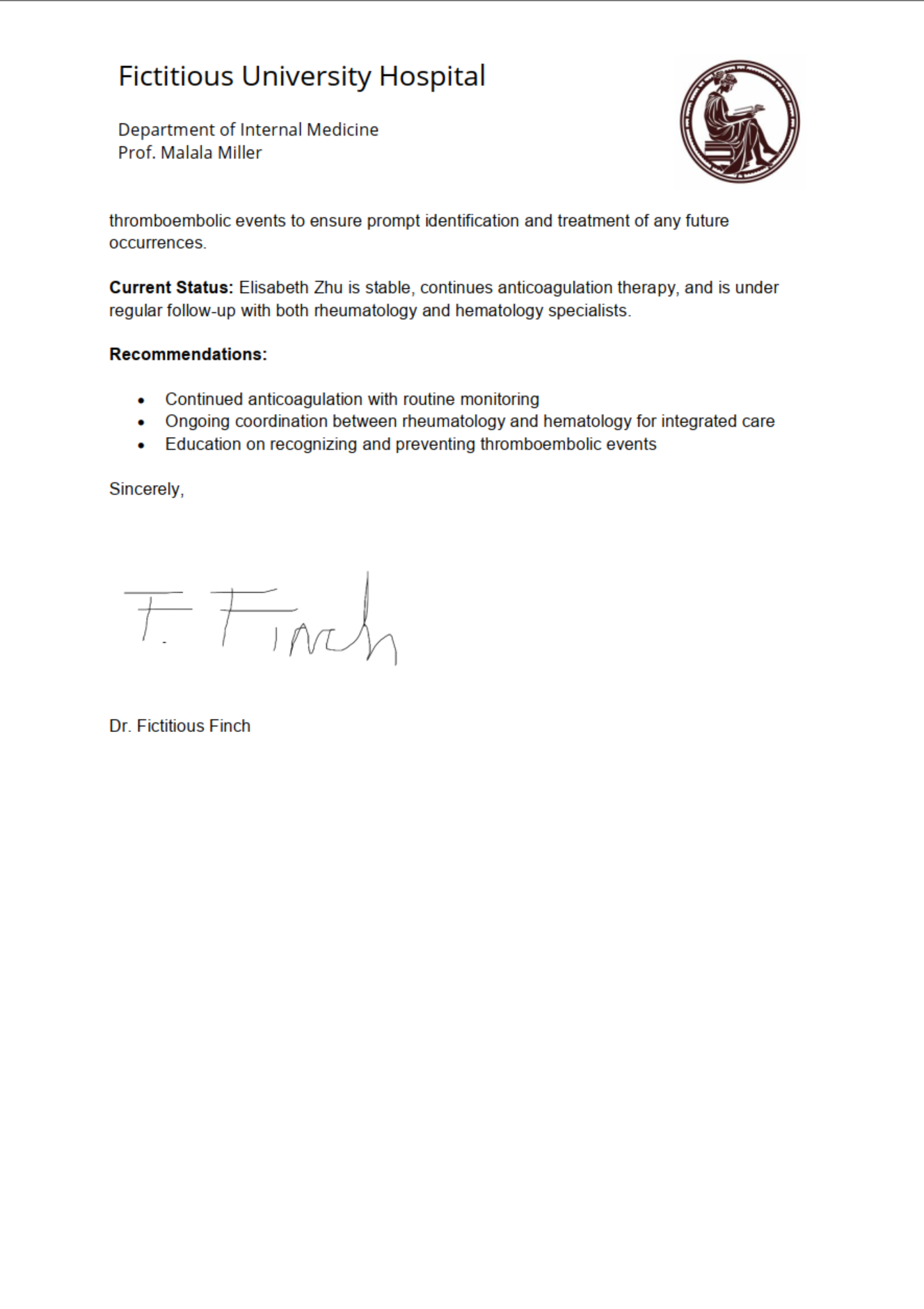


### Fictitious Examples Redacted
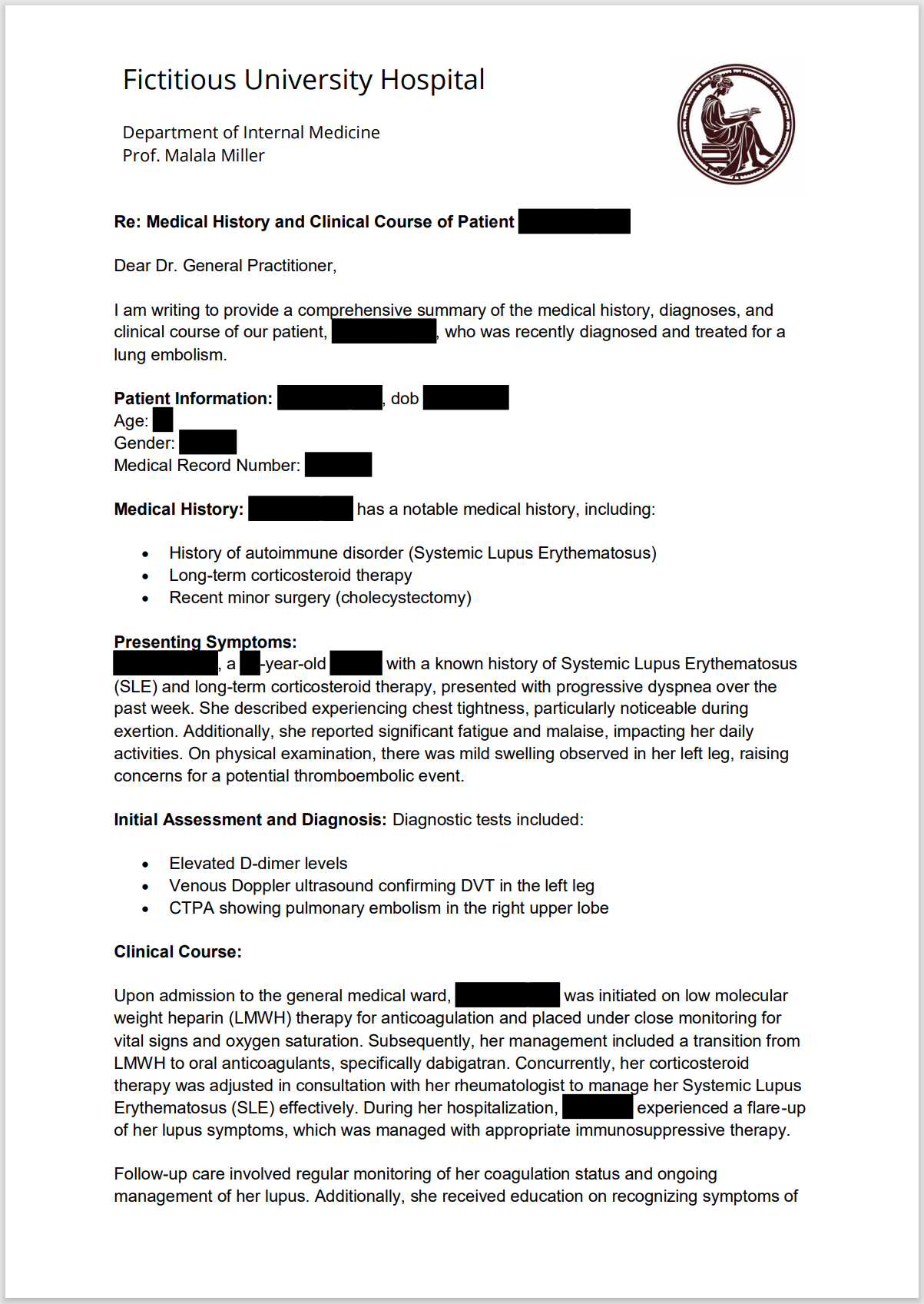


##
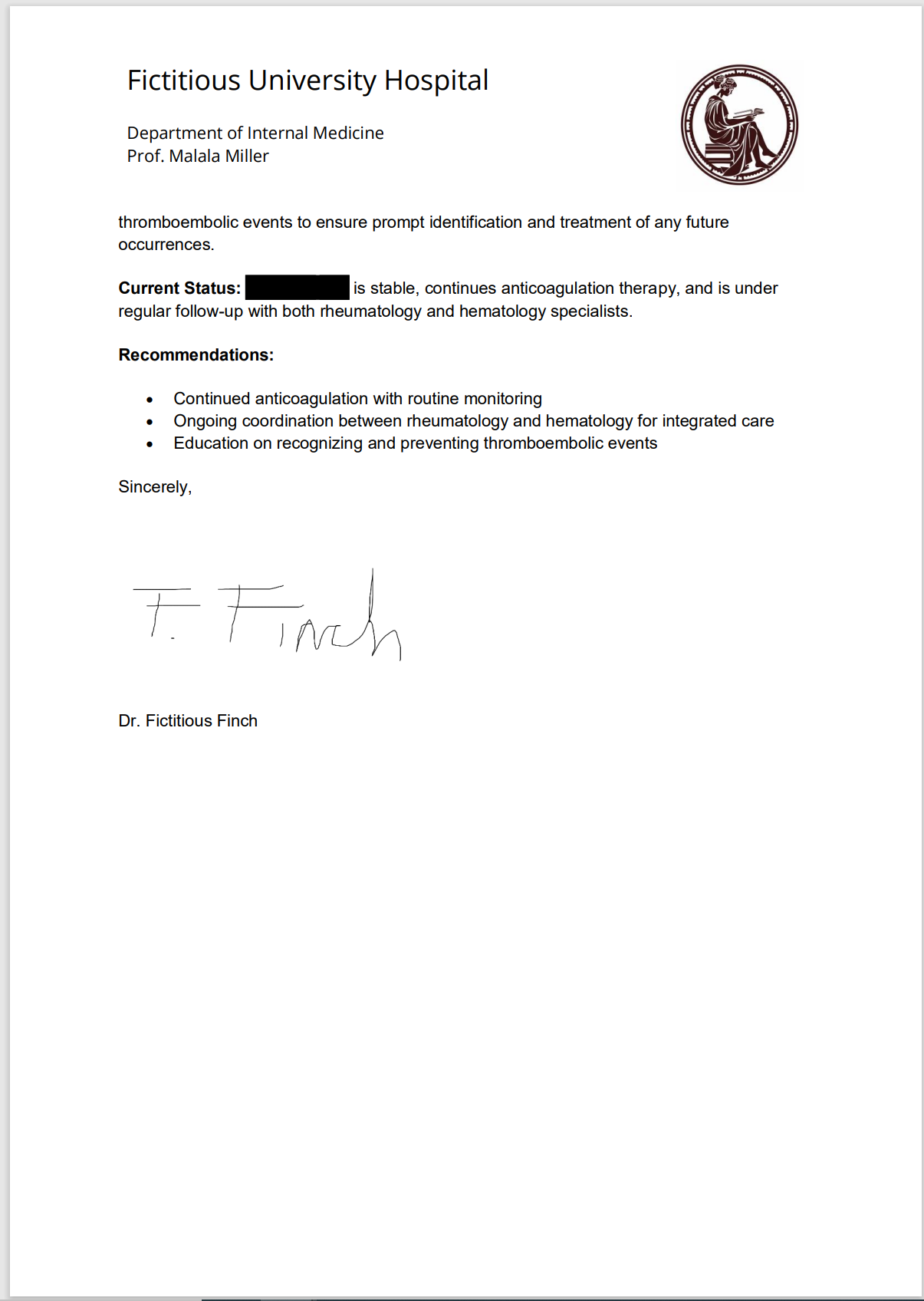


We present the redacted clinical letter from one out of eight fictitious examples here. The downloaded, redacted PDFs contain blackened identifiers as defined by the user. The text layer behind is completely removed.

## Guide through pulmonary embolism example
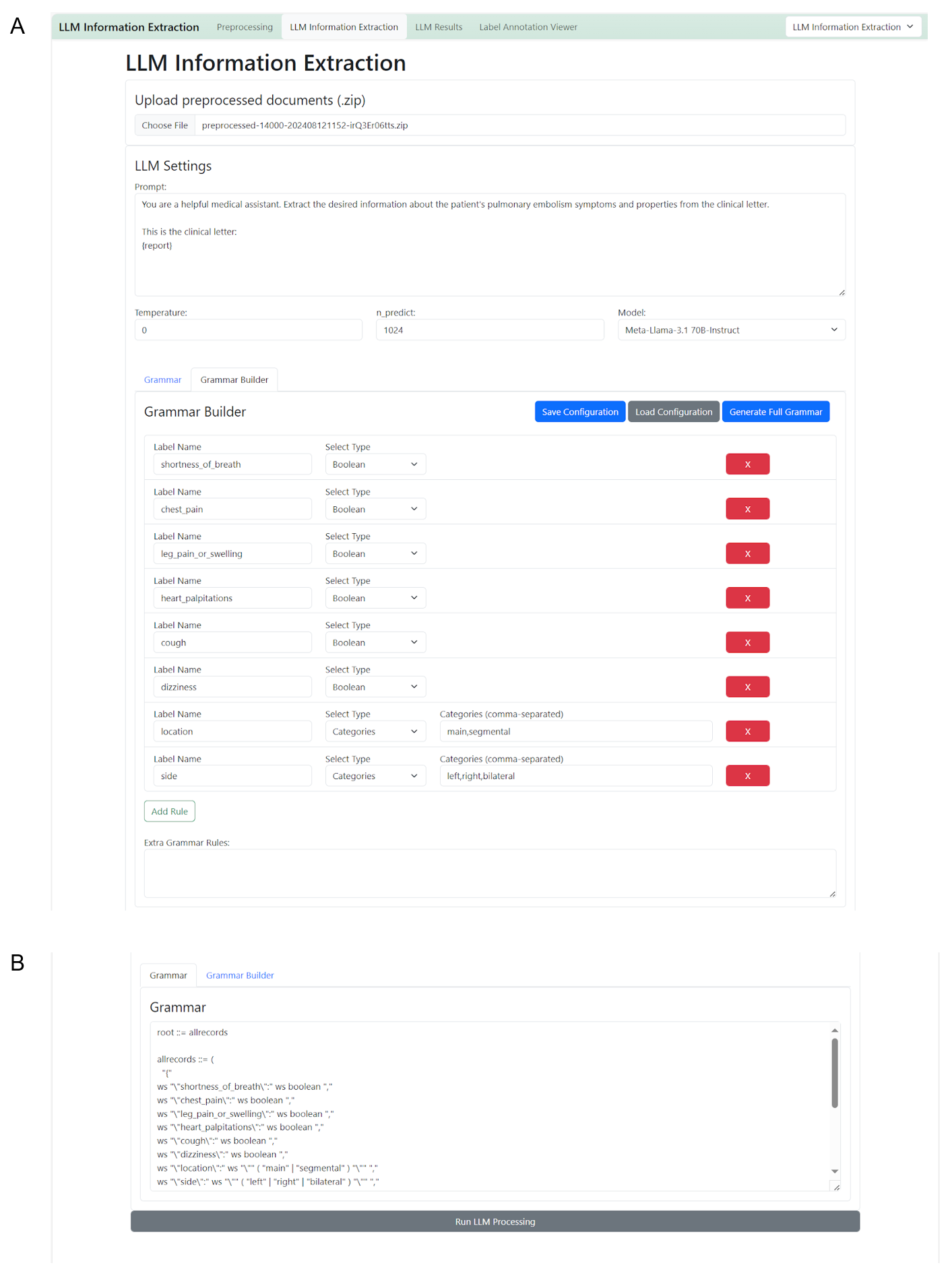


### **Supplementary Figure 6**

Preparation for LLM based information extraction for pulmonary embolism symptoms. We defined a set of symptoms as well as the location of the pulmonary embolism as variables to extract from the 8 fictitious clinical letters and built a grammar with the grammar builder tool (**A**) and then generated and adopted in the “Grammar” field (**B**) . The prompt was very simple and is given in the “Prompt” field. The model used was Meta’s Llama 3.1 70B in 4-bit quantization and GGUF format available from “huggingface”.


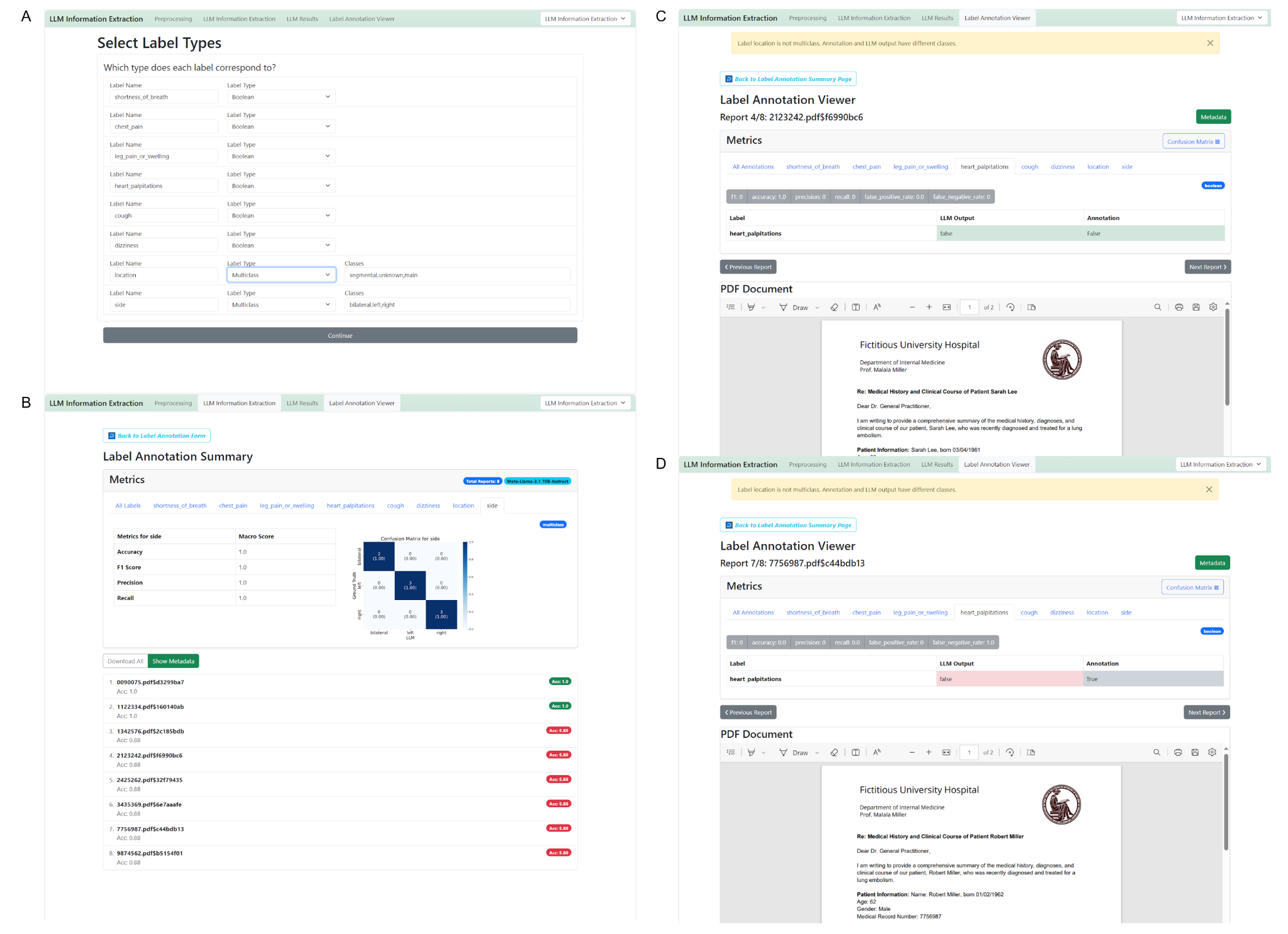


### **Supplementary Figure 7**

Evaluation of Information Extraction. **A** For information extraction tasks, it is essential to confirm the data types of variables. Boolean variables enable the use of more diverse metrics than string variables, which are limited to simple string matching. When defining multiclass variables, their characteristics should be provided in a comma-separated format.**B** The annotation summary presents metrics for all variables collectively and for each variable individually. For categorical and boolean variable types, confusion matrices are shown alongside a table that includes comprehensive metrics such as accuracy, precision, recall, and F1 score. The results for all variables can be reviewed on a per-document basis. **C** An example of a correctly identified clinical letter, where the patient complains about palpitations. **D** An example of an incorrect output from the language model (LLM) for the symptom of palpitations.

## Guide through experiment 2

###
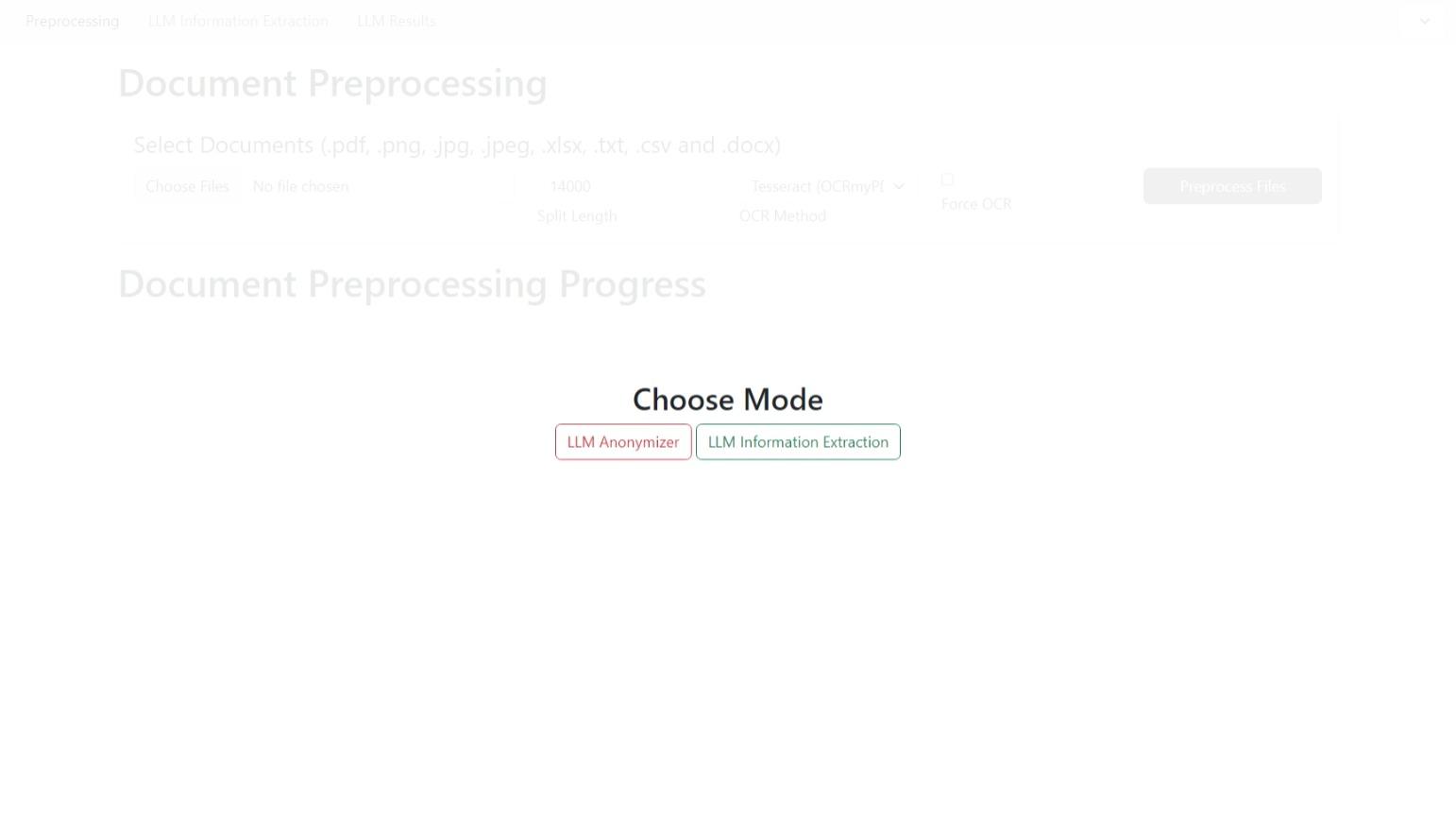
**Supplementary Figure 8**

Choose Mode. Depending on the task, in this case information extraction, the mode can be chosen when starting the process but also switched flexibly during the process.

###
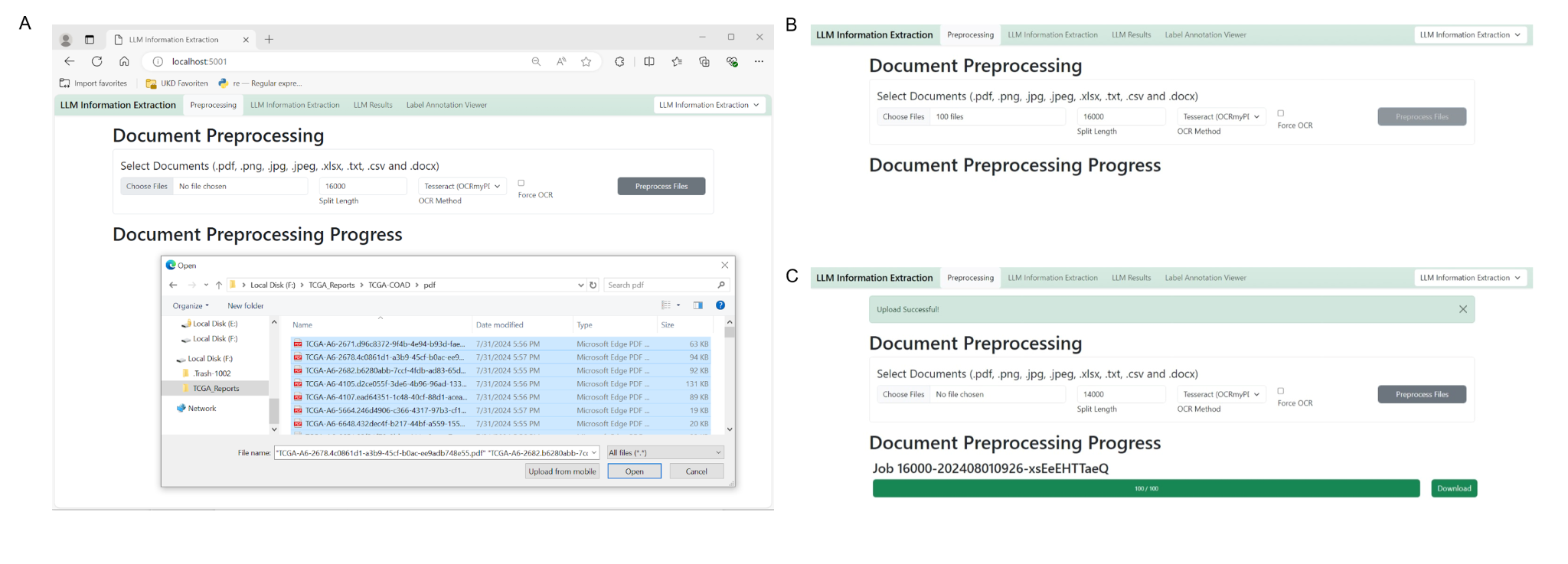


### **Supplementary Figure 9**

Data preprocessing for TCGA reports. All 100 TCGA reports in portable document format (PDF) were uploaded simultaneously. The preprocessing process was initiated with the “Preprocess files” button and a zip file containing the preprocessed documents could be downloaded. The PDFs already contained a text layer, therefore preprocessing without optical character recognition (OCR) took place.


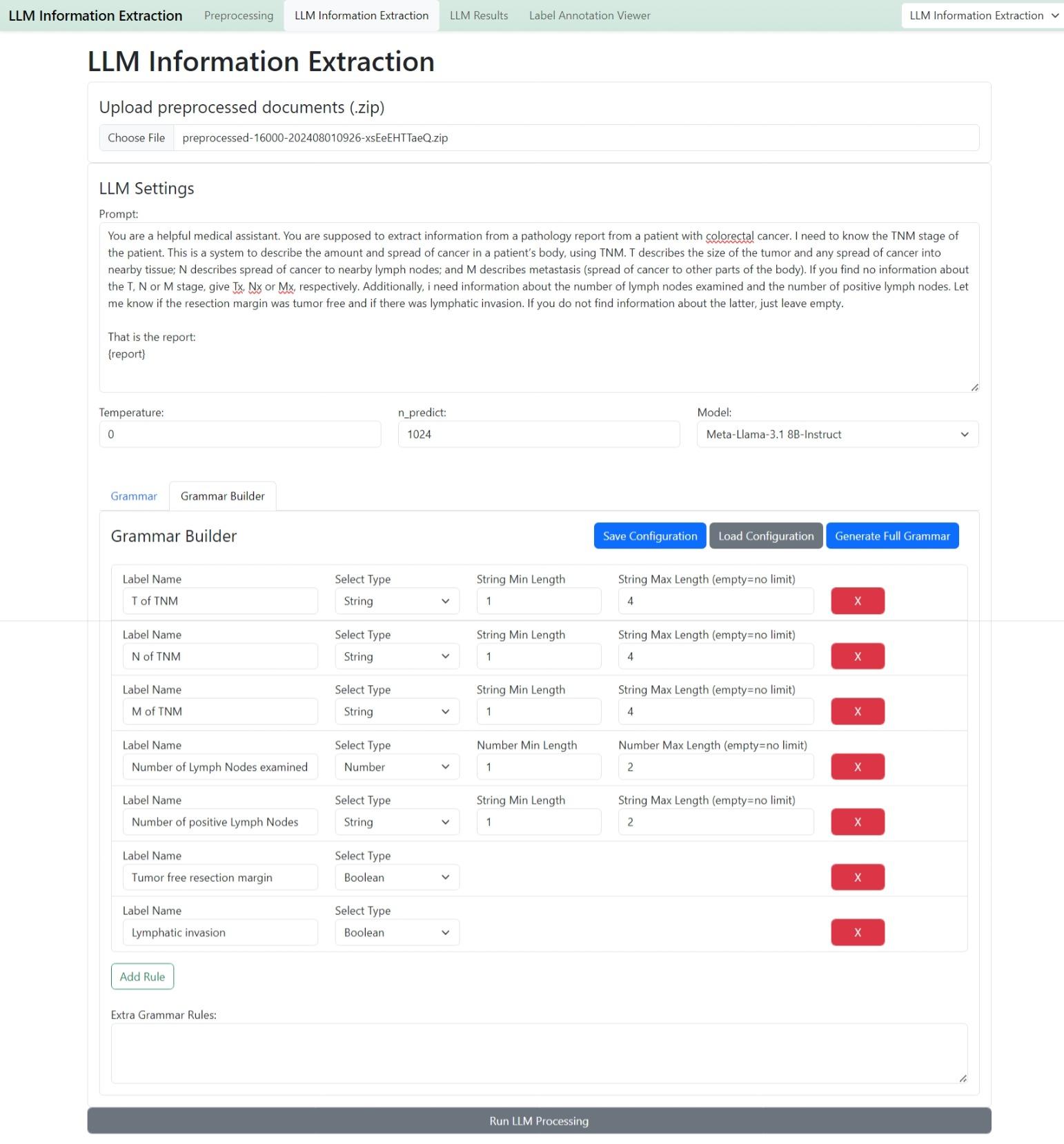


### **Supplementary Figure 10**

LLM Information Extraction. The preprocessed zip file of all 100 TCGA reports was uploaded. Afterwards, the prompt was defined as specified in the figure and model as well as hyperparameters were chosen. The grammar builder assisted for defining the grammar. Initially, T-, N-, and M-stage were defined as strings, “Number of lymph nodes examined” and “-positive” were defined as numbers and the variables “Tumor free resection margin” and “lymphatic invasion" were defined as boolean variables.

## Troubleshooting Examples


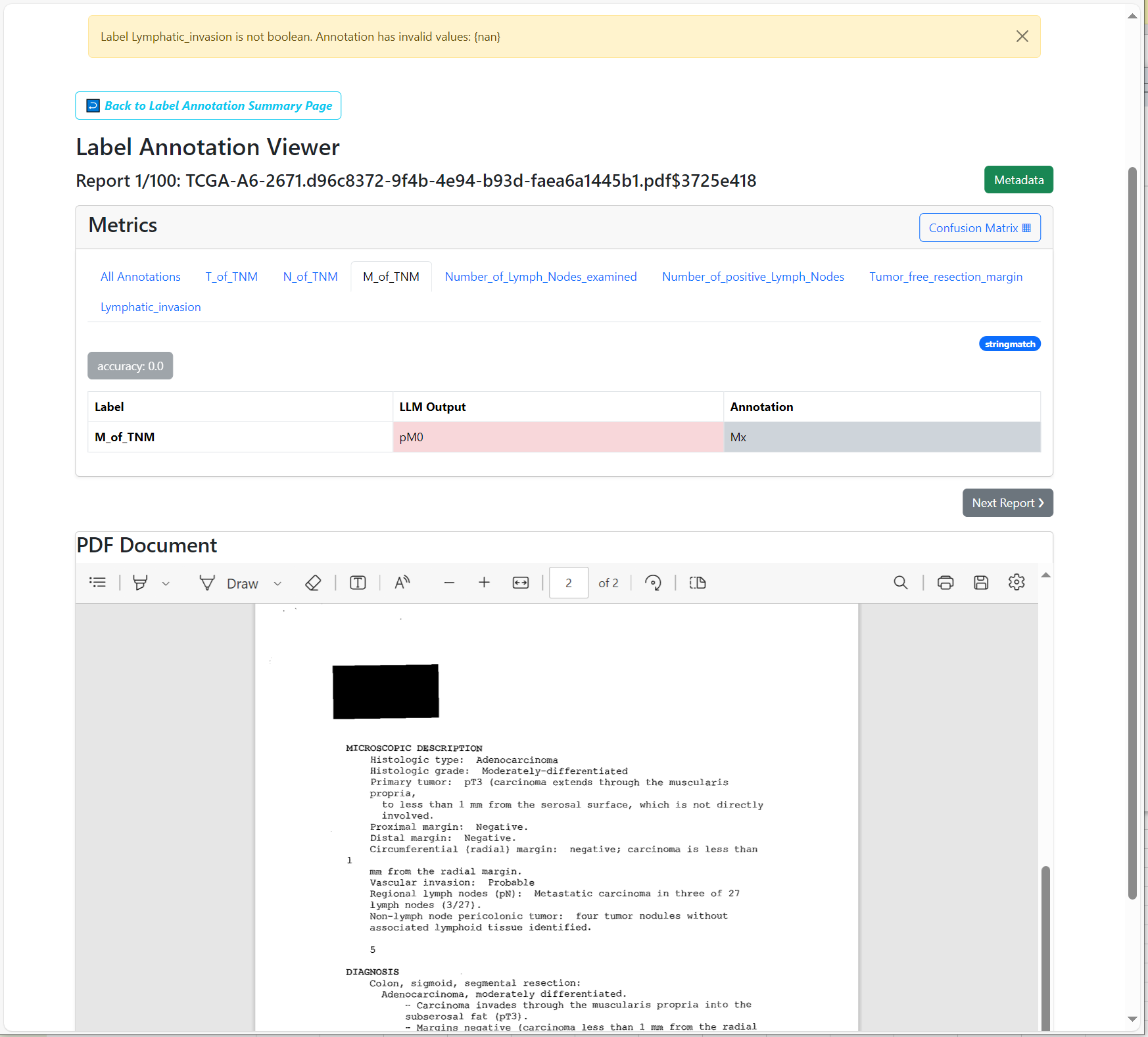


### **Supplementary Figure 11**

Model Hallucination. There is no information in the pathology report about the metastatic status of the tumor. Therefore, the correct answer would be “Mx”, but the model outputs pM0.


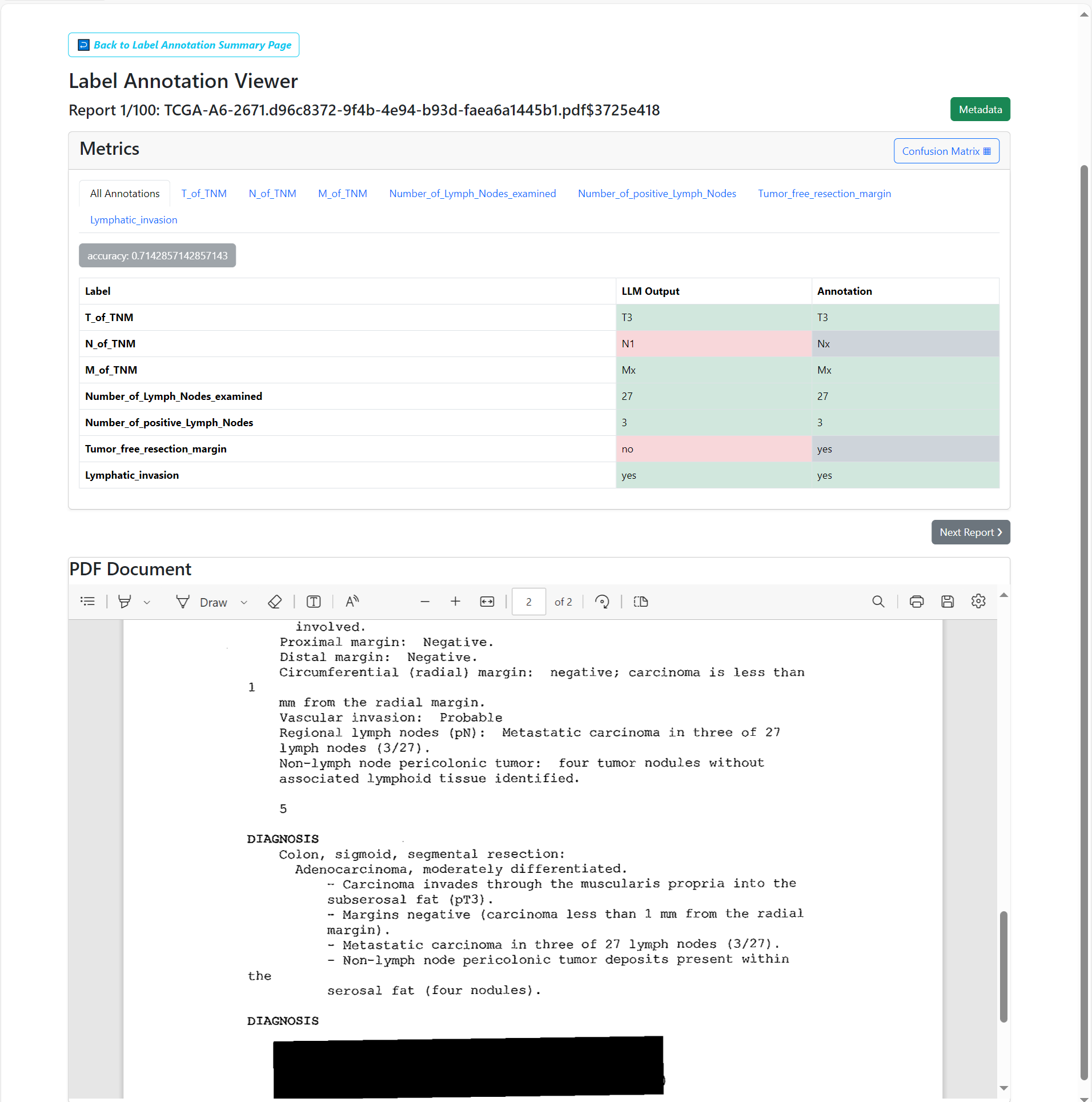


### **Supplementary Figure 12**

Conflicting input text data, Example 1. For colorectal cancer, negative margins in a pathology report refer to the fact that the resection margins are tumor-free. The report states that “margins [are] negative”, but if the carcinoma is located less than 1mm distant from the resection margin, this is accompanied by bad prognosis and therefore could be considered as “margins positive”. In this case, a consensus needed to be made in order to define the absolute ground truth.


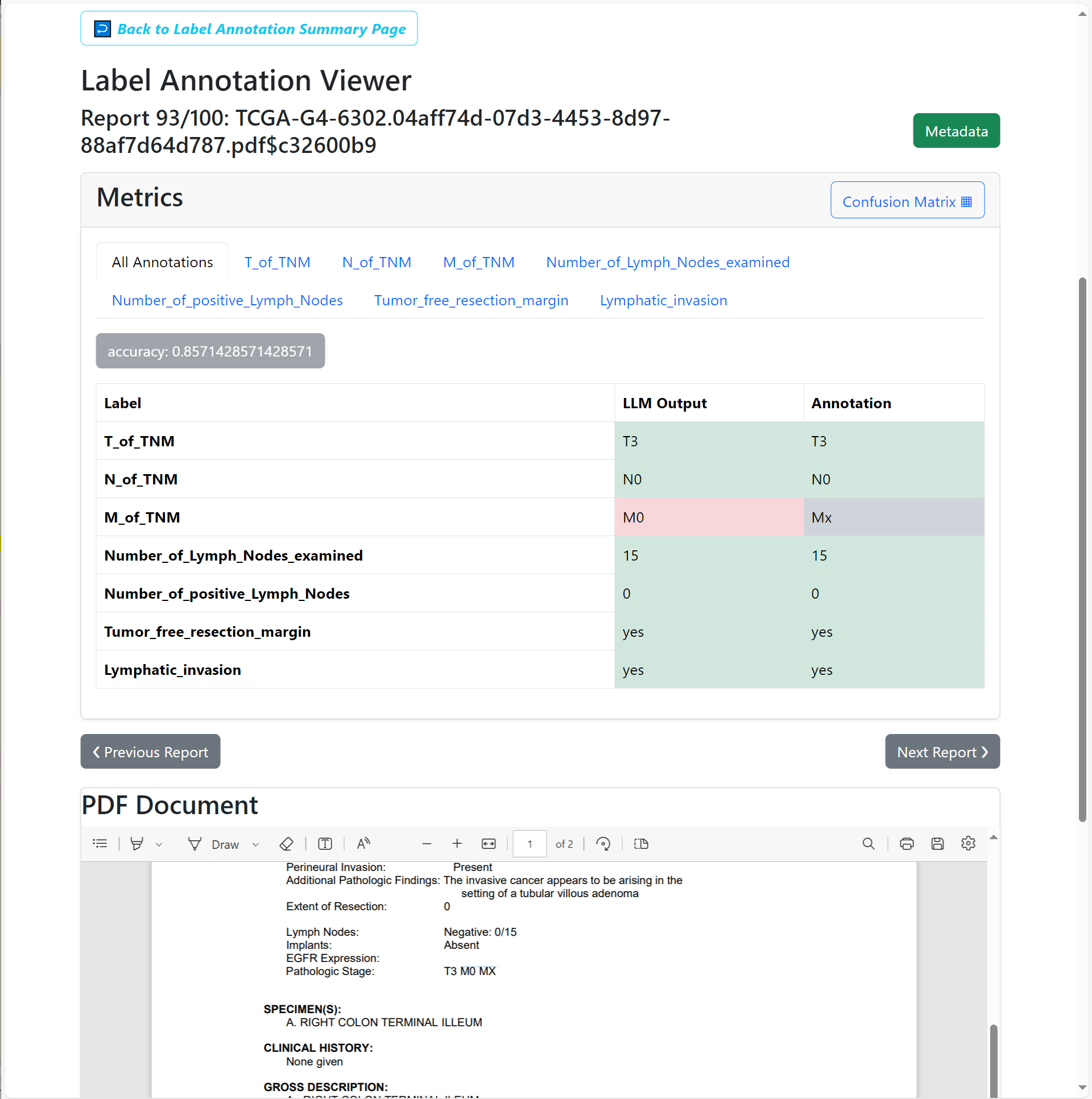


### **Supplementary Figure 13**

Conflicting Input text data, Example 2. The pathologic stage section gives conflicting information about the metastasis status by stating “T3 M0 Mx”. In this report, the only specimen examined is colorectal tumor tissue, there is no information about metastasis, therefore MX should be the correct answer. The LLM extracts “M0”, which is also conflictingly stated in the document.


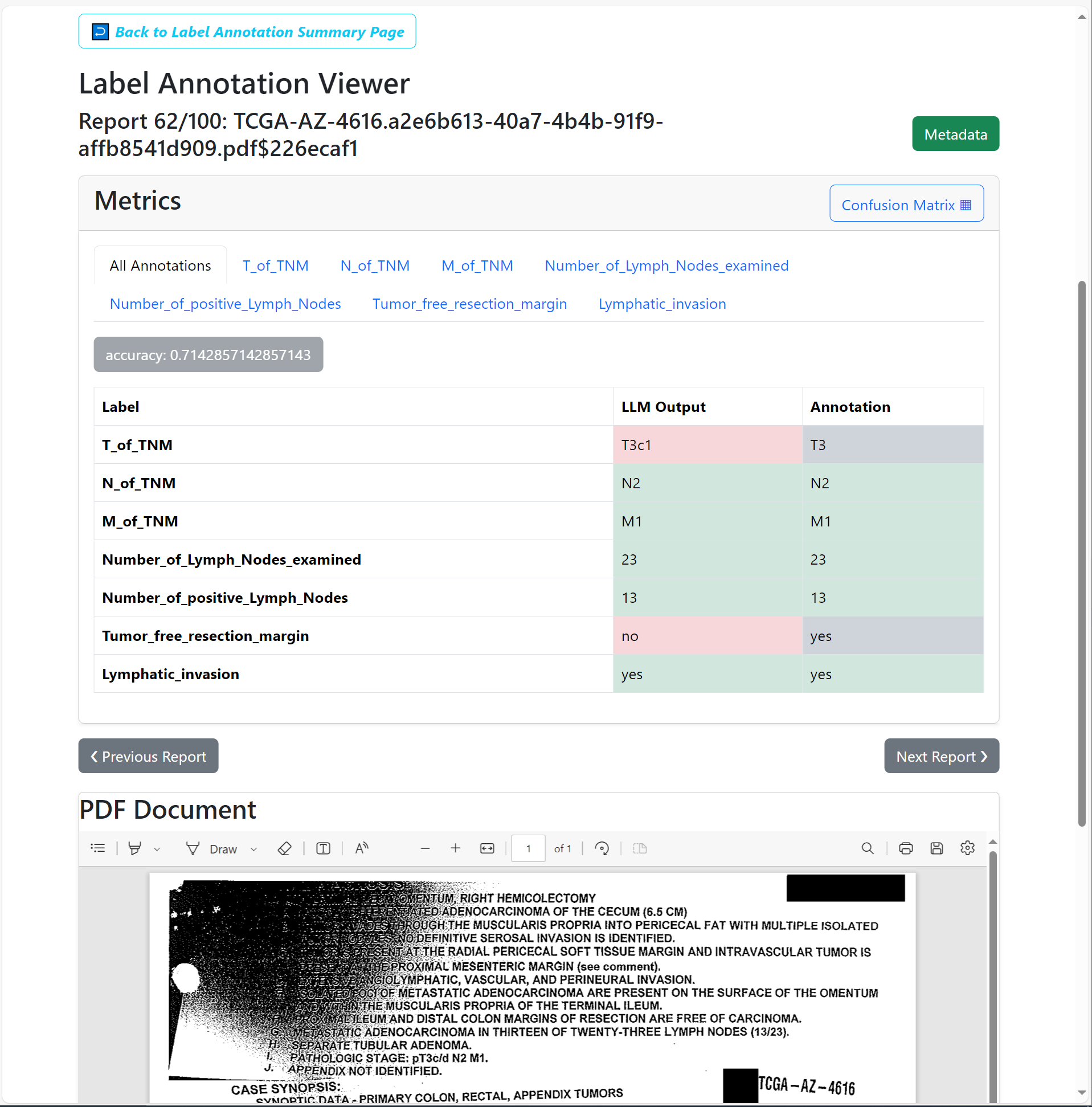


### **Supplementary Figure 14**

Annotation is coarser than LLM. The LLm extracts the described T-stage from the pathology report, which is “T3c1”, which is more detailed than the annotation which only provides “T3”, therefore detected as mismatching, even though both are correct.


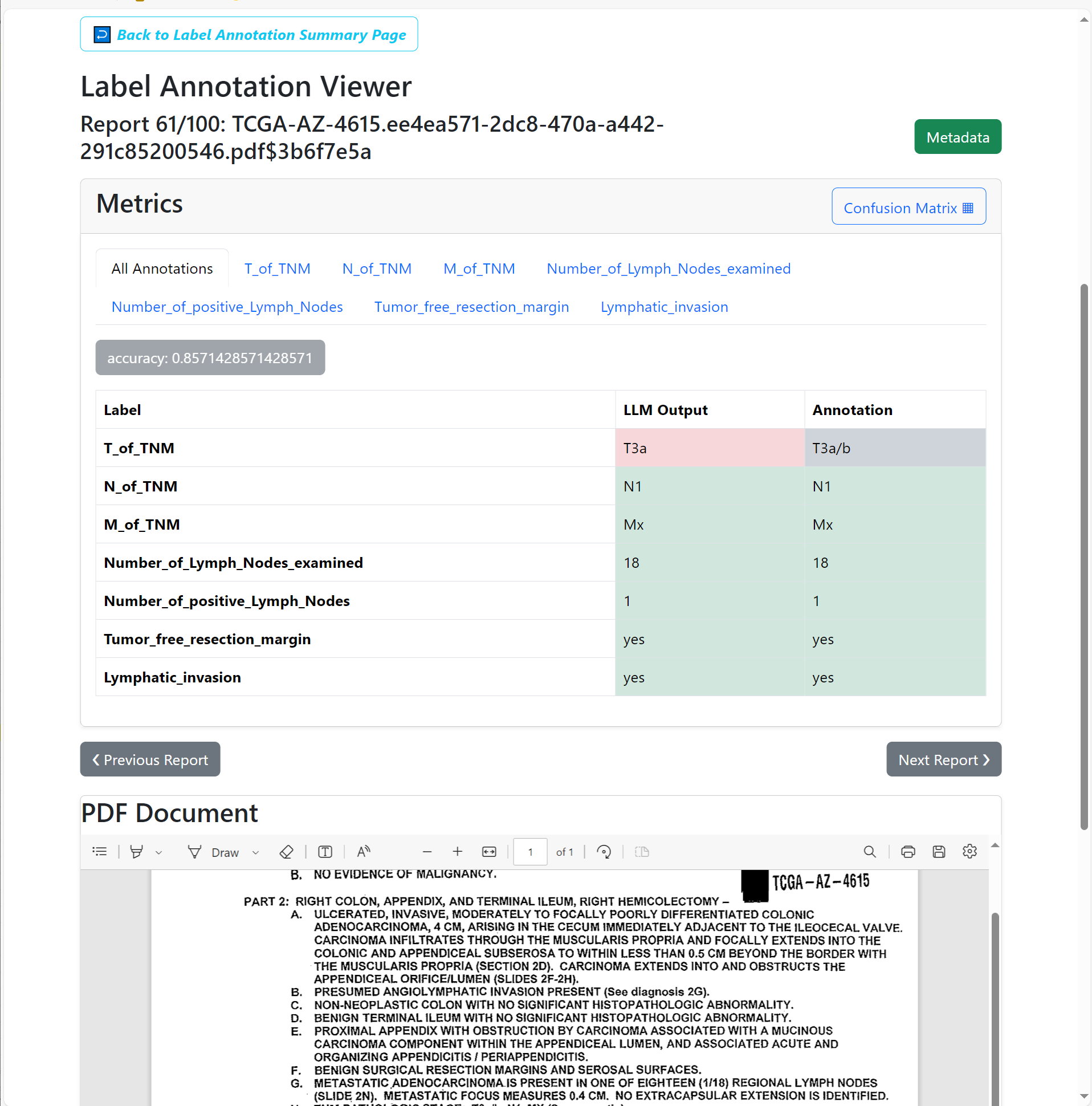


### **Supplementary Figure 15**

Annotation is more detailed than LLM. The annotation contained a more detailed description of the T-stage than the LLM-output and suggests that definite classification to T3a or T3b was not possible. The LLM output was T3a, which is slightly different but not entirely incorrect when compared to the ground truth.

###
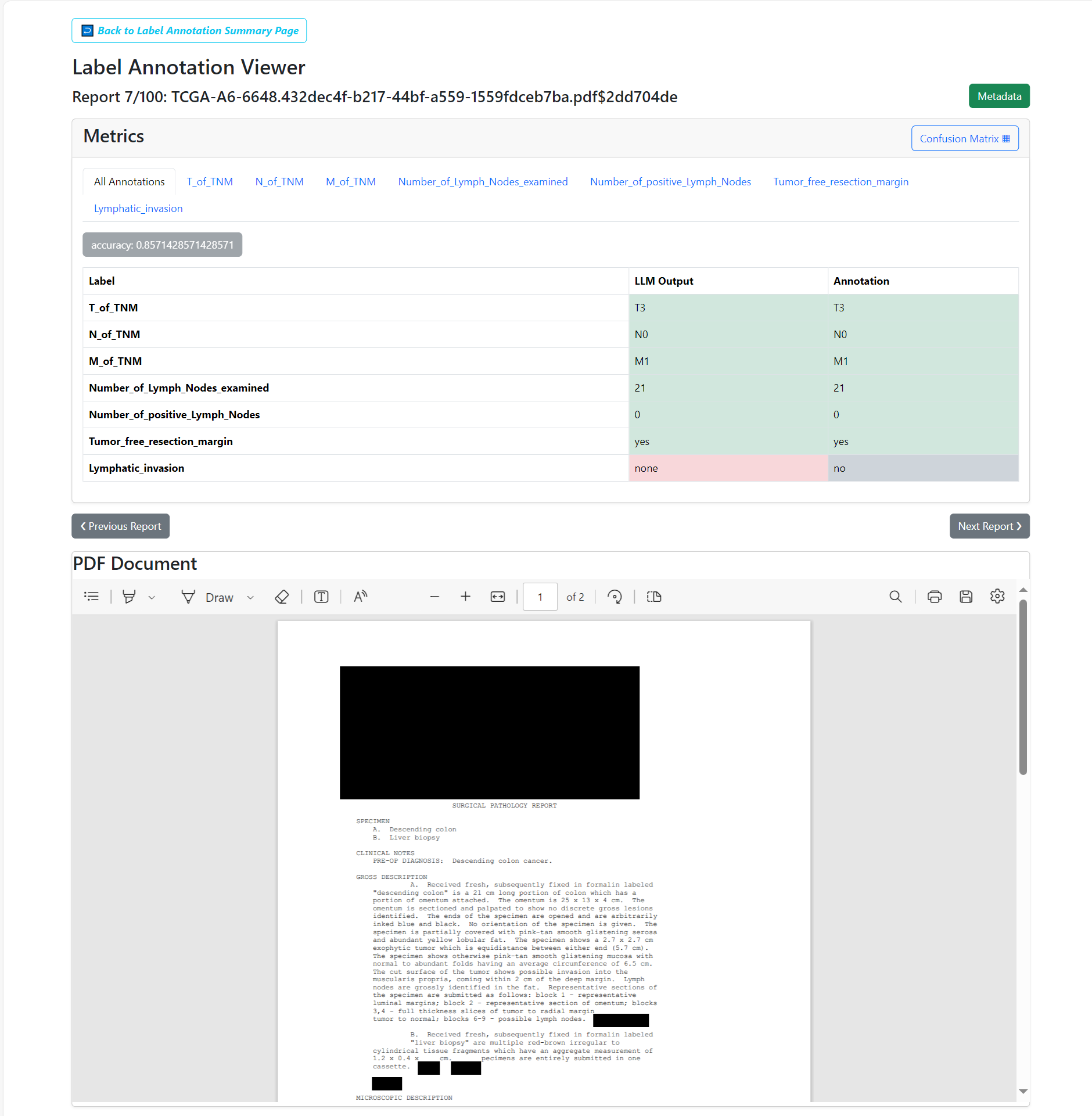


### **Supplementary Figure 16**

Output classes lack sufficient distinguishability. In this example, the presence of lymphatic invasion needs to be extracted from TCGA pathology reports of colorectal cancer specimens. Three answer options are given to the LLM: “yes”, ”no”, and “none”, if there is no information at all about lymphatic invasion. These three classes lack sufficient distinguishability in natural language, as “no” and “none” are semantically too close. Improvement can be achieved by defining classes that are more distinguishable in natural language, such as “yes”, “no”, “not mentioned”.


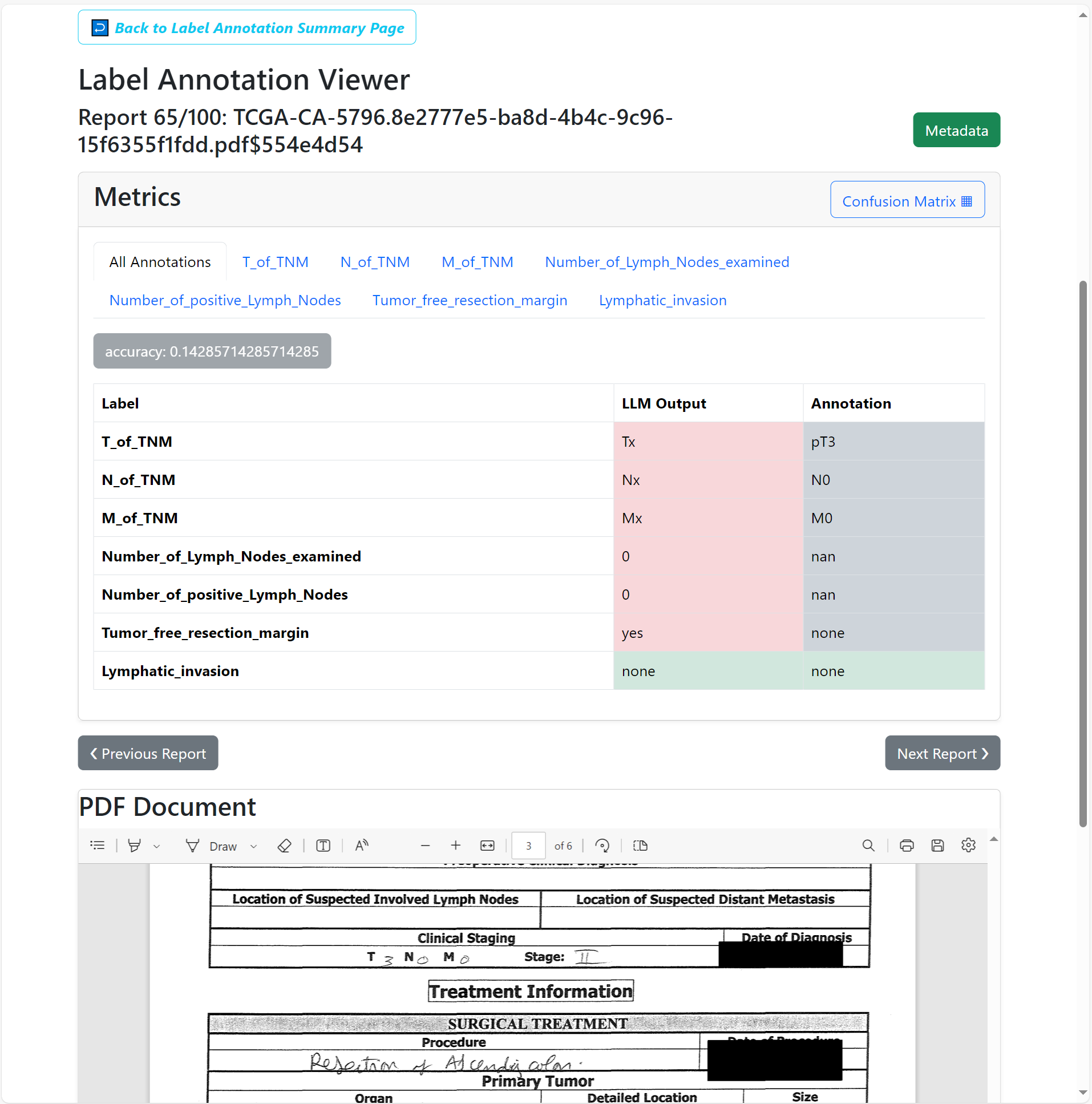


### **Supplementary Figure 17**

Failure in information extraction due to bad input data quality. When documents contain a high proportion of handwritten information and PDFs are of very poor quality, text extraction may fail due to data preprocessing. This failure can be addressed by choosing an alternative OCR method for preprocessing. The "Surya" OCR method generally outperforms "Tesseract," and trOCR, paddleOCR and visionLLM models such as Phi are superior in detecting handwritten text.


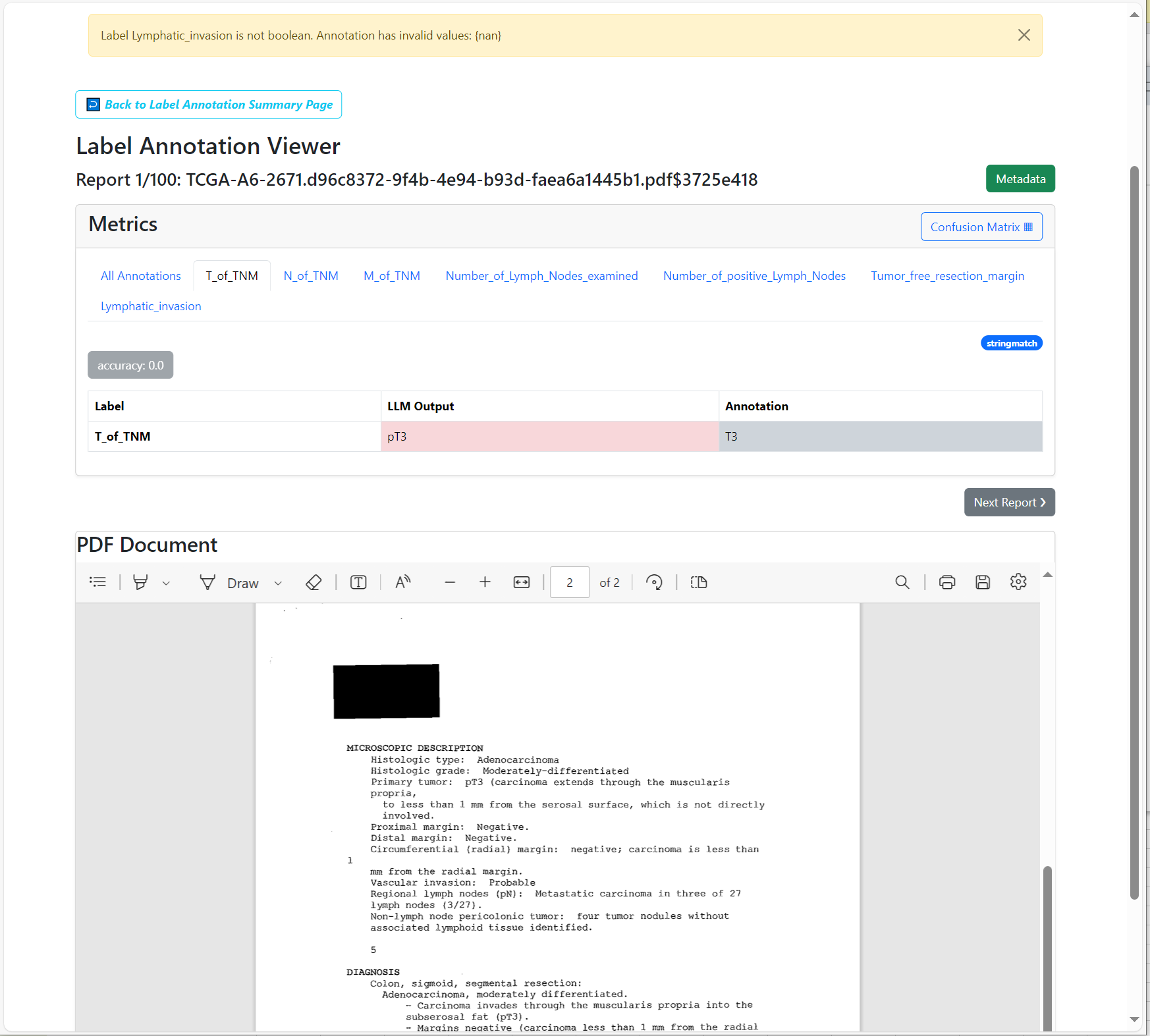


### **Supplementary Figure 18**

Accurate LLM output with minor character discrepancies compared to the annotation. The report states a “pT3” tumor stage, as the T-stage was defined through histopathological examination of the specimen. The annotation confirms that T3 is correct, but the “p” was not relevant and therefore omitted by the ground truth rater. This discrepancy can be solved via prompt engineering: The desired output needs to be specified as detailed as possible within the prompt and grammar.


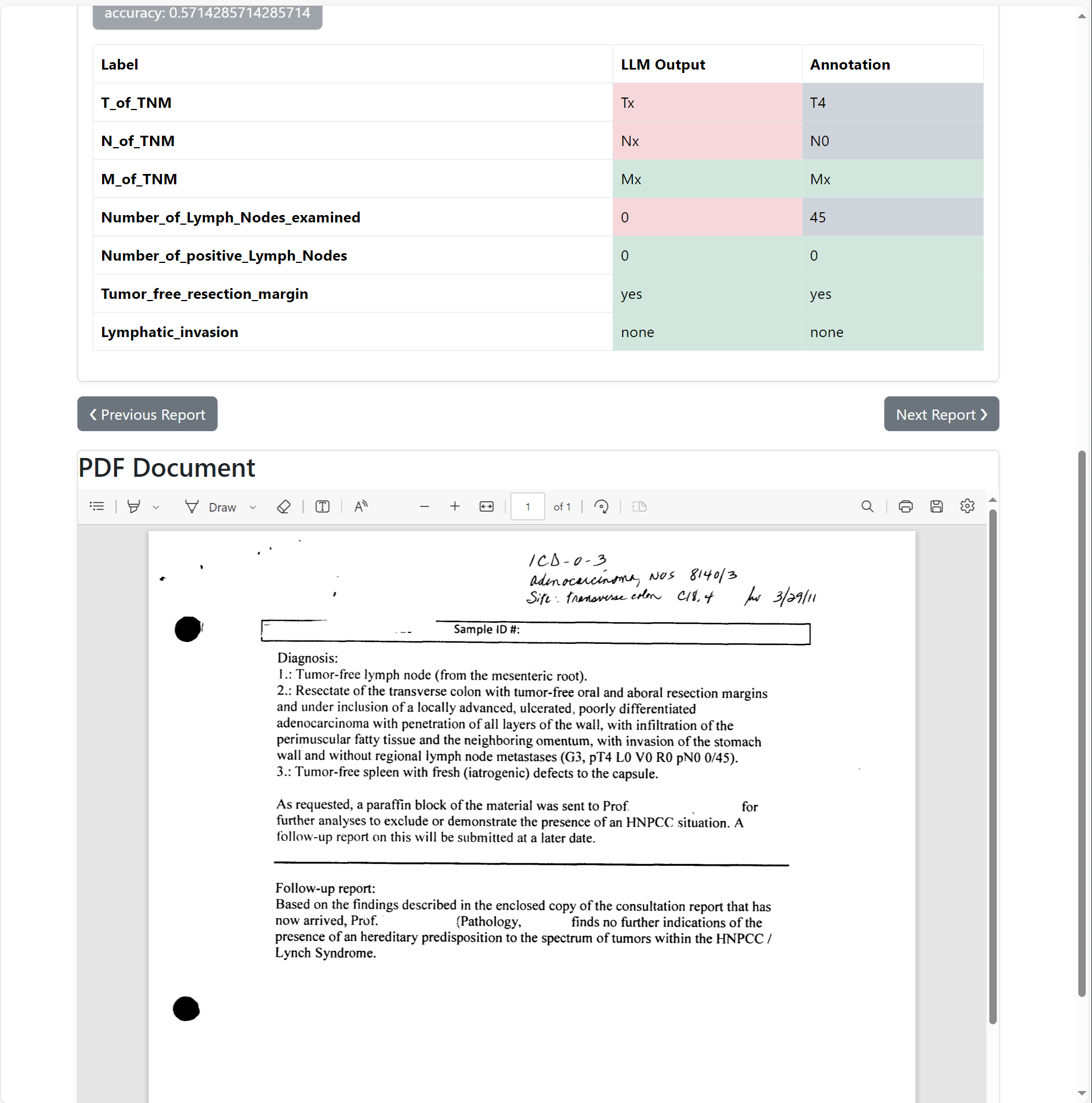


### **Supplementary Figure 19**

Information is present but could not be detected by the LLM. In this example, the whole tumor formula was given in the text as “G3, pT4 L0 V0 R0 pN0 0/45”. However, this information could not be extracted by the LLM. This could be solved by adding a more detailed explanation of how the tumor formula looks like and few-shot examples in the prompt.


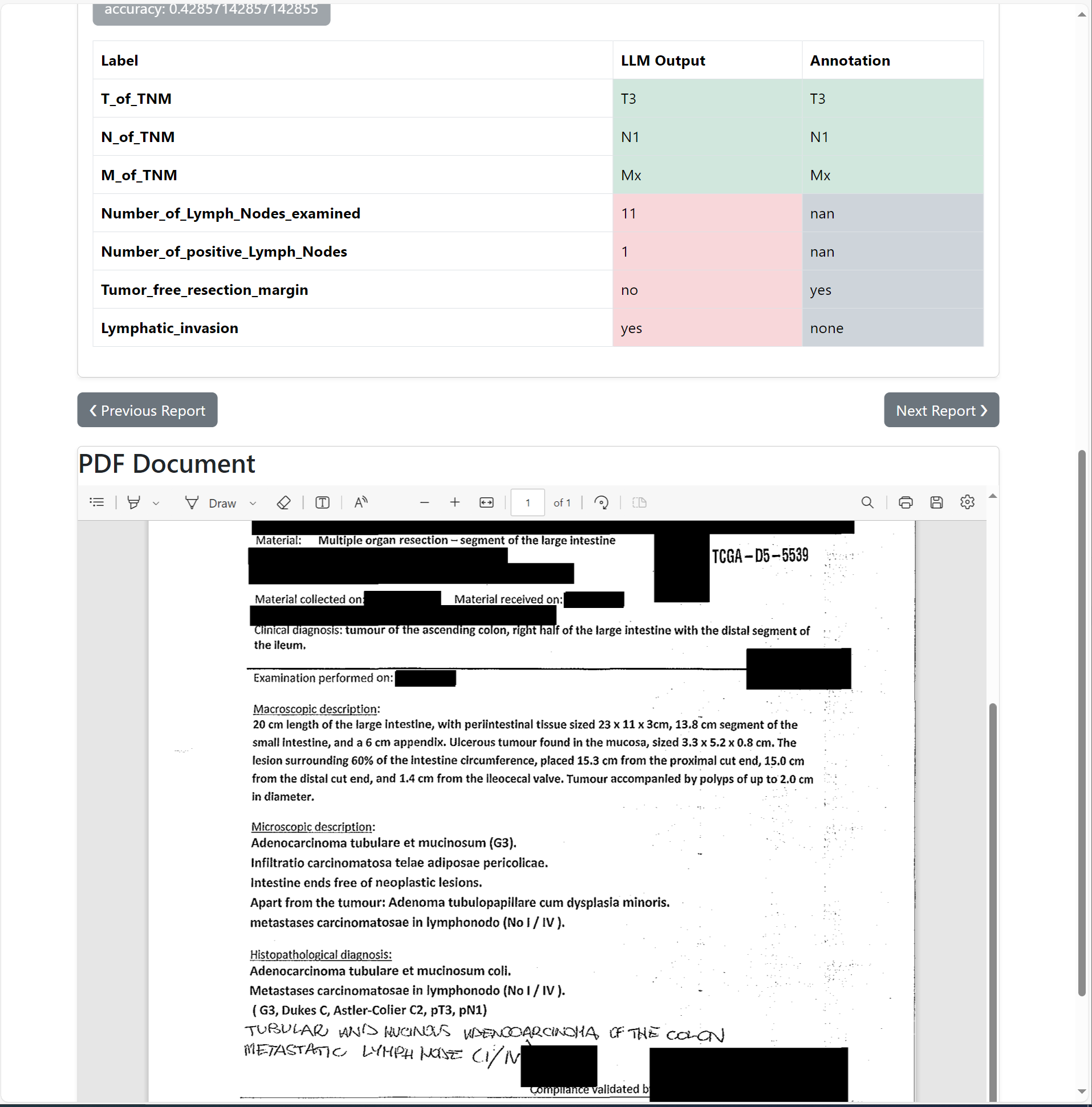


### **Supplementary Figure 20**

LLM detects more than human rater. In this case, the report mentions “Metastases carcinomatosae in lymphonodo (No I/IV)." This information is also noted in handwriting as “metastatic lymph node (I/IV)." The LLM correctly identifies that one lymph node is positive out of the examined lymph nodes. However, the Roman numeral IV was incorrectly extracted as 11 lymph nodes were examined. This error stems from OCR, where the Roman numeral IV was misinterpreted as II.


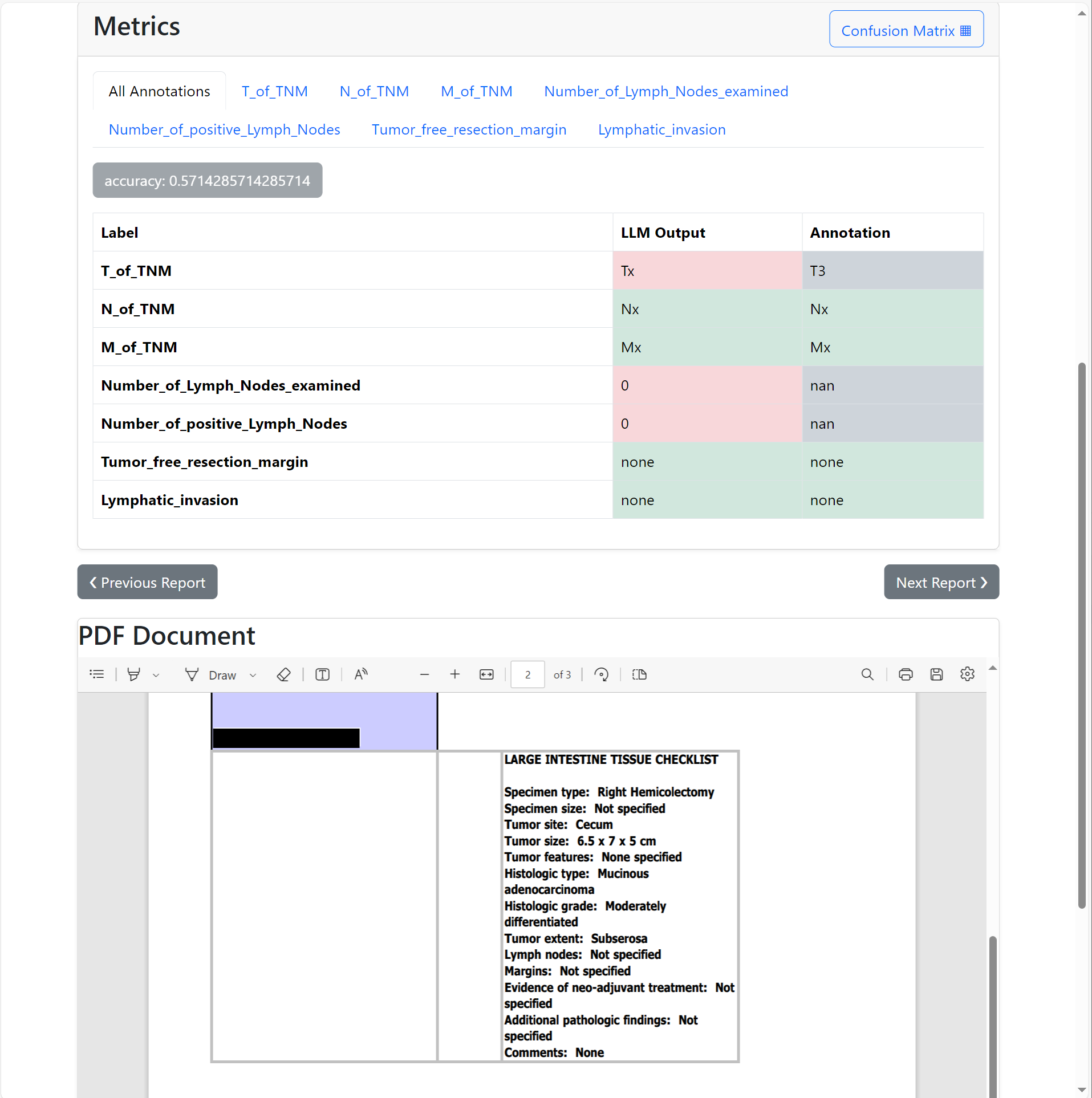


### **Supplementary Figure 21**

LLM lacks implicit knowledge. The T-stage describes the size and extent of the tumor. The report mentions that tumor extent reaches the subserosa, which corresponds to T3. This was not detected by the LLM and can be solved through more detailed information in the prompt and few-shot examples.


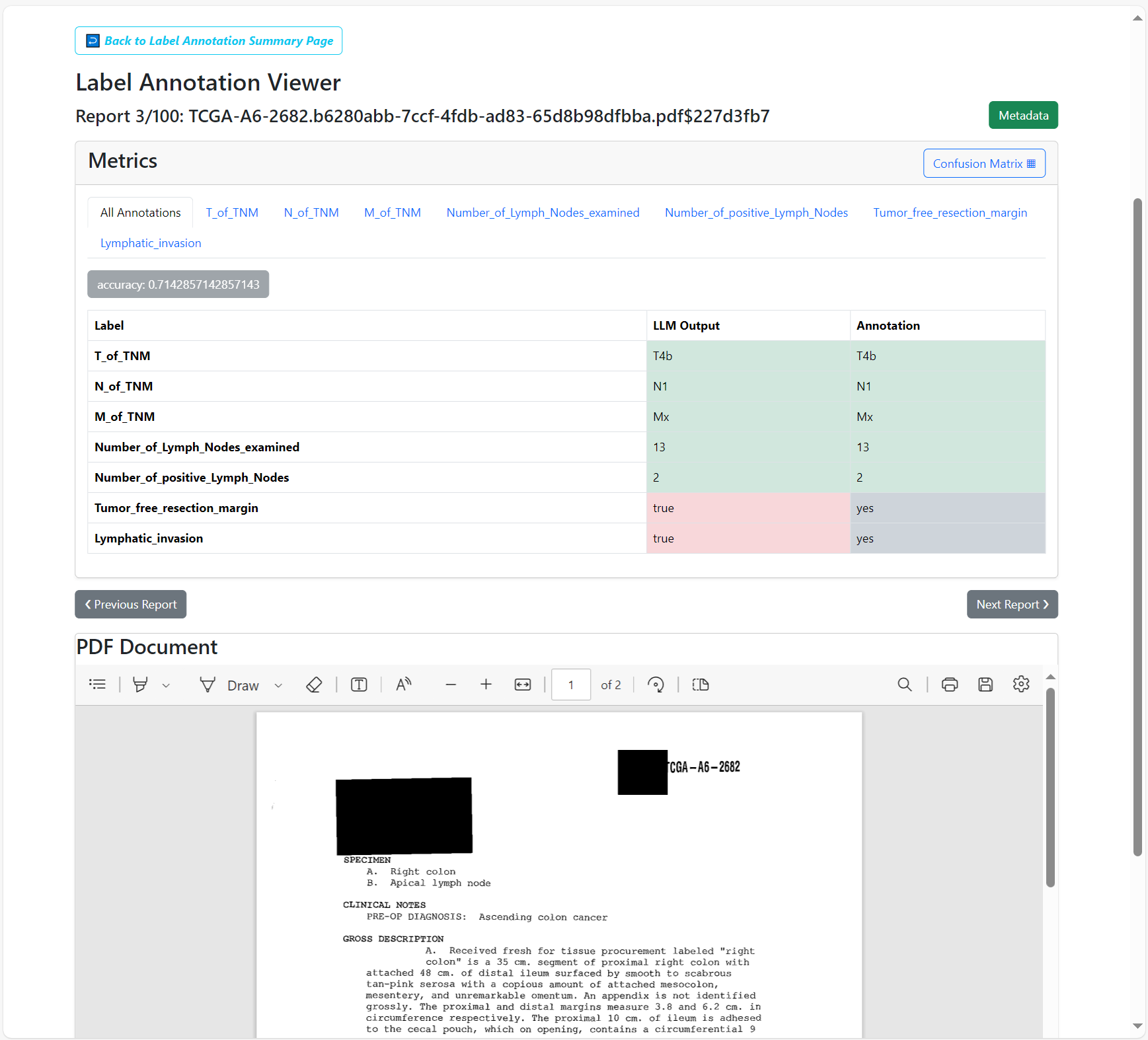


### **Supplementary Figure 22**

Mismatch in output categories. The output categories must be defined exactly as they appear in the annotated ground truth table. Otherwise, the LLM output might be correct ("true"), but if the ground truth says "yes," it will be considered a mismatch despite having the same meaning.

57. Neves M, Ševa J. An extensive review of tools for manual annotation of documents. *Brief Bioinform*. 2021;22(1):146-163.

58. Waltl B, Bonczek G, Matthes F. Rule-based information extraction: Advantages, limitations, and perspectives. Published online 2018. https://wwwmatthes.in.tum.de/file/47fs4e04rvtp/Sebis-Public-Website/-/Rule-based-Information-Extraction-Advantages-Limitations-and-Perspectives/Wa18b.pdf

59. Freitag D. Machine Learning for Information Extraction in Informal Domains. *Mach Learn*. 2000;39(2):169-202.

60. Banko M, Moore RC. Part-of-Speech Tagging in Context. In: *COLING 2004: Proceedings of the 20th International Conference on Computational Linguistics*. COLING; aug 23--aug 27 2004:556-561.

61. Naradowsky J, Riedel S, Smith D. Improving NLP through Marginalization of Hidden Syntactic Structure. In: Tsujii J ’ichi, Henderson J, Paşca M, eds. *Proceedings of the 2012 Joint Conference on Empirical Methods in Natural Language Processing and Computational Natural Language Learning*. Association for Computational Linguistics; 2012:810-820.

62. Khazaei T, Xiao L. Corpus-based analysis of rhetorical relations: A study of lexical cues. In: *Proceedings of the 2015 IEEE 9th International Conference on Semantic Computing (IEEE ICSC 2015)*. IEEE; 2015:417-423.

63. Pritam K. Advancements and methodologies in Natural Language Processing and machine learning: A comprehensive review. *Int J Res Appl Sci Eng Technol*. 2024;12(4):1495-1500.

64. Nagarhalli TP, Vaze V, Rana NK. Impact of Machine Learning in Natural Language Processing: A Review. In: *2021 Third International Conference on Intelligent Communication Technologies and Virtual Mobile Networks (ICICV)*. IEEE; 2021:1529-1534.

65. Brown P, Pietra VD, Souza PD, Lai J, Mercer R. Class-Based n-gram Models of Natural Language. *CL*. 1992;18:467-479.

66. Ando R, Zhang T. A framework for learning predictive structures from multiple tasks and unlabeled data. *J Mach Learn Res*. 2005;6:1817-1853.

67. Blitzer J, McDonald RT, Pereira FC. Domain adaptation with structural correspondence learning. *Empir Method Nat Lang Process*. Published online July 22, 2006:120-128.

68. Mikolov T, Sutskever I, Chen K, Corrado G, Dean J. Distributed Representations of Words and Phrases and their Compositionality. *Adv Neural Inf Process Syst*. Published online October 16, 2013:3111-3119.

69. Pennington J, Socher R, Manning C. GloVe: Global Vectors for Word Representation. In: Moschitti A, Pang B, Daelemans W, eds. *Proceedings of the 2014 Conference on Empirical Methods in Natural Language Processing (EMNLP)*. Association for Computational Linguistics; 2014:1532-1543.

70. Howard J, Ruder S. Universal Language Model Fine-tuning for Text Classification. *arXiv [csCL]*. Published online January 18, 2018. http://arxiv.org/abs/1801.06146

71. Biswas R, De S. A Comparative Study on Improving Word Embeddings Beyond Word2Vec and GloVe. In: *2022 Seventh International Conference on Parallel, Distributed and Grid Computing (PDGC)*. IEEE; 2022:113-118.

72. Vaswani A, Shazeer N, Parmar N, et al. Attention is all you need. *Adv Neural Inf Process Syst*. 2017;30. https://proceedings.neurips.cc/paper/7181-attention-is-all

73. Perez-Lopez R, Ghaffari Laleh N, Mahmood F, Kather JN. A guide to artificial intelligence for cancer researchers. *Nat Rev Cancer*. 2024;24(6):427-441.

74. Patwardhan N, Marrone S, Sansone C. Transformers in the Real World: A Survey on NLP Applications. *Information*. 2023;14(4):242.

75. Lewis P, Ott M, Du J, Stoyanov V. Pretrained Language Models for Biomedical and Clinical Tasks: Understanding and Extending the State-of-the-Art. In: *Proceedings of the 3rd Clinical Natural Language Processing Workshop*. Association for Computational Linguistics; 2020:146-157.

76. Wang B, Xie Q, Pei J, et al. Pre-trained Language Models in Biomedical Domain: A Systematic Survey. *ACM Comput Surv*. 2023;56(3):1-52.

77. Dagdelen J, Dunn A, Lee S, et al. Structured information extraction from scientific text with large language models. *Nat Commun*. 2024;15(1):1418.

78. Alkhalaf M, Yu P, Yin M, Deng C. Applying generative AI with retrieval augmented generation to summarize and extract key clinical information from electronic health records. *J Biomed Inform*. 2024;156:104662.
